# Detailed Expression Pattern of Aldolase C (Aldoc) in the Cerebellum, Retina and Other Areas of the CNS Studied in Aldoc-Venus Knock-In Mice

**DOI:** 10.1371/journal.pone.0086679

**Published:** 2014-01-27

**Authors:** Hirofumi Fujita, Hanako Aoki, Itsuki Ajioka, Maya Yamazaki, Manabu Abe, Arata Oh-Nishi, Kenji Sakimura, Izumi Sugihara

**Affiliations:** 1 Department of Systems Neurophysiology, Tokyo Medical and Dental University Graduate School, Bunkyo-ku, Tokyo, Japan; 2 Center for Brain Integration Research, Tokyo Medical and Dental University Graduate School, Bunkyo-ku, Tokyo, Japan; 3 Department of Cellular Neurobiology, Brain Research Institute, Niigata University, Niigata, Japan; 4 Molecular Neuroimaging Program, Molecular Imaging Center, National Institute of Radiological Sciences, Inage-ku, Chiba, Japan; 5 Systems Neurobiology Laboratory, The Salk Institute for Biological Studies, La Jolla, California, United States of America; 6 Department of Otolaryngology-Head and Neck Surgery, The Johns Hopkins University School of Medicine, Baltimore, Maryland, United States of America; Institut de la Vision, France

## Abstract

Aldolase C (Aldoc, also known as “zebrin II”), a brain type isozyme of a glycolysis enzyme, is expressed heterogeneously in subpopulations of cerebellar Purkinje cells (PCs) that are arranged longitudinally in a complex striped pattern in the cerebellar cortex, a pattern which is closely related to the topography of input and output axonal projections. Here, we generated knock-in Aldoc-Venus mice in which Aldoc expression is visualized by expression of a fluorescent protein, Venus. Since there was no obvious phenotypes in general brain morphology and in the striped pattern of the cerebellum in mutants, we made detailed observation of Aldoc expression pattern in the nervous system by using Venus expression in Aldoc-Venus heterozygotes. High levels of Venus expression were observed in cerebellar PCs, cartwheel cells in the dorsal cochlear nucleus, sensory epithelium of the inner ear and in all major types of retinal cells, while moderate levels of Venus expression were observed in astrocytes and satellite cells in the dorsal root ganglion. The striped arrangement of PCs that express Venus to different degrees was carefully traced with serial section alignment analysis and mapped on the unfolded scheme of the entire cerebellar cortex to re-identify all individual Aldoc stripes. A longitudinally striped boundary of Aldoc expression was first identified in the mouse flocculus, and was correlated with the climbing fiber projection pattern and expression of another compartmental marker molecule, heat shock protein 25 (HSP25). As in the rat, the cerebellar nuclei were divided into the rostrodorsal negative and the caudoventral positive portions by distinct projections of Aldoc-positive and negative PC axons in the mouse. Identification of the cerebellar Aldoc stripes in this study, as indicated in sample coronal and horizontal sections as well as in sample surface photos of whole-mount preparations, can be referred to in future experiments.

## Introduction

Aldolase is an enzyme involved in one of the essential steps in glycolysis, a process required in all cells that consume glucose. Aldolase also plays several non-glycolytic roles, including interactions with vacuolar-H^+^-ATPase and other molecules [1]–[6]. The three isozymes of aldolase, aldolases A, B and C, are expressed predominantly in the muscle and in the brain, in the liver, and in the brain, respectively [7], [8]. Neuronal expression of Aldoc has only been reported so far in the cerebellum and retina.

Aldoc ( = zebrin II) has long been used as a marker for the study of cerebellar compartmentalization, due to its specific expression in distinct subpopulations of cerebellar Purkinje cells (PCs) that are arranged in functionally significant longitudinal stripe-shaped compartments. Each longitudinal compartment determined by the striped Aldoc expression pattern (Aldoc compartments) is innervated by climbing fibers originating from a specific subarea of the inferior olive [9]–[13], and by mossy fibers of different sources to a certain extent [14]–[16]. PCs in each Aldoc compartment then project to a specific subarea of the cerebellar nuclei [17]. In addition, expression patterns of many molecules are closely related to that of Aldoc [18] in the PC population; for example, phospholipase Cβ4 (PLCB4) is expressed in a complementary pattern to that of Aldoc [19]. Moreover, PCs in Aldoc-positive and -negative compartments have different physiological properties [20], [21]. These features of Aldoc compartments are generally consistent between individuals and are preserved across mammalian species from rodents to primates [18], [22]–[27]. Thus, Aldoc compartments appear to reflect a basic organization of the cerebellar cortex.

The visualization of Aldoc expression with fluorescence through gene manipulation will be of great use to anatomical and physiological studies of cerebellar compartmentalization, because the location of cerebellar Aldoc stripes can be clearly and easily identified without immunostaining, even in physiological *in vivo* and *in vitro* preparations. However, transgenic mice produced by using specific promoters of *Aldoc* gene failed to reproduce its expression pattern in PCs [28], [29]. Here, we produced a knock-in mouse strain in which a gene of mutated green fluorescent protein (Venus, [30]) was inserted to the exon 2 of the *Aldoc* gene. In this mouse strain, the knocked-in Venus gene should be expressed under the same transcriptional control as the *Aldoc* gene.

In the present study, we first examined Venus expression, which is supposed to mirror Aldoc expression, throughout the CNS, including the retina in the heterozygous Aldoc-Venus mouse. Then, after confirming that the cerebellar striped expression pattern of Venus in the heterozygote was virtually the same as that of Aldoc in the wild type mouse, the striped expression pattern of Venus was carefully examined throughout the cerebellar cortex by applying serial section alignment analysis (SSAA) in coronal, horizontal and parasagittal sections in the heterozygote. The stripes of cerebellar Aldoc/Venus expression were fully re-identified. Indeed, we clarified previously unknown striped patterns in the flocculus. The results are summarized in a schematic mapping of stripes on the unfolded cerebellar cortex and are also indicated on sample photomicrographs of the cerebellar sections and outer aspects, which may be referred to as an atlas for future studies.

## Methods

### Generation of Aldoc-Venus Knock-in Mice

All animal experiments were carried out in accordance with the guidelines of the animal welfare committee of the Tokyo Medical and Dental University and Niigata University, subsequent to approval by the Ethics Review Committee for Animal Experimentation of Tokyo Medical and Dental University and Niigata University (approval number 0130211A, Tokyo Medical and Dental Univ., and 178-8, Niigata Univ.).

**Figure 1:**
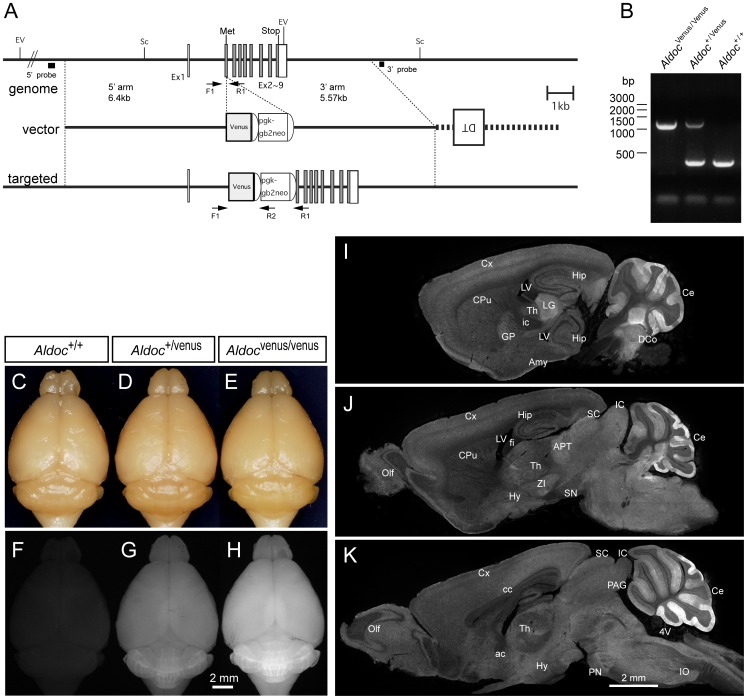
Generation of Aldoc-Venus knock-in mice and Venus expression in their brain. A, Schematic representations of Aldoc genome, targeting vector and targeted genome. Black boxes indicate exons. Black boxes indicate the probes for Southern blot analysis. Semicircles indicate FRT sequences. Met, initial methionine; DT, diphtheria toxin gene; Venus, Venus gene; pgk-gb2neo, neomycin-resistant gene expression cassette; Sc, ScaI; EV, EcoRV. B, PCR genotyping of mice tail DNA detects 383 bp band from the wile-type allele and 1169 bp band from the targeted allele. C-H, Bright field illumination (C–E) and epifluorescence (F–H) photomicrographs of the whole brain of adult littermates (75-day old) bred from heterozygous parents (C and F, wild type; D and G, heterozygote; E and H, homozygote). I–K. Epifluorescence photomicrographs of sagittal sections of the brain of a heterozygote. Scale bar in K applies to I–K. Abbreviations for anatomical terms in this and following figures: 3V, third ventricle; 4V, forth ventricle; I–VI, layer I–VI; I–X, lobules I–X; a–c, sublobules a–c (as in VIa); ac, anterior commissure; AIN, anterior interposed nucleus; Amy, amygdala; APT, anterior pretectal nucleus; beta, subnucleus beta; C, caudal; cc, corpus callosum; Ce, cerebellum; Cop, copula pyramidis; CPu, caudate and putamen; Cr I, crus I of the ansiform lobule; CF, climbing fiber; Cr II, crus II of the ansiform lobule; Cx, cerebral cortex; D, dorsal; DAO, dorsal accessory olive; DC, dorsal cap of Kooy; DCo, dorsal cochlea nucleus; DH, dorsal horn; DLH, dorsolateral hump (of the AIN); DLP, dorsolateral protuberance (of the MN); EPl, external plexiform layer (olfactory bulb); fi, fimbria of hippocampus; Fl, flocculus; GCL, ganglion cell layer; gl, granule cell layer (cerebellum); GP, globus pallidus; GrO, granular cell layer of olfactory bulb; Hip, hippocampus; Hy, hypothalamus; ic, internal capsule; IC, inferior colliculus; INL, inner nuclear layer (retina); IO, inferior olive; IPl, internal plexiform layer (olfactory bulb); L, lateral; LMol, lacunosum moleculare layer; LN, lateral nucleus; Lt, left; LV, lateral ventricle; M, medial; MAO, medial accessory olive; ml, molecular layer (cerebellum); MN, medial nucleus; Olf, olfactory bulb; ONL, outer nuclear layer (retina); Or, oriens layer of hippocampus; PAG, periaqueductal gray; Par, paramedian lobule; pcl, PC layer; pf, primary fissure; PFl, paraflocculus; PIN, posterior interposed nucleus; PN, pontine nucleus; Py, pyramidal cell layer of hippocampus; R, rostral; Rad, stratum radiatum of hippocampus; Rt, right; SC, superior colliculus; Sim, simple lobule; SN, substantia nigra; Th, thalamus; V, ventral; VH, ventral horn; ZI, zona incerta.

To generate the Aldoc-Venus knock-in mouse, we designed a targeting vector in which the Venus [30] gene was placed just behind the translational initiation site of the *Aldoc* gene in frame. The knock-in vector pAldocVnsTV contained a 6.40 kb fragment at the 5′ side, a Venus gene placed behind the Aldoc translational start, a pgk-1 promoter-driven neomycin phosphotransferase gene (neo) flanked by two Flp recognition target (frt) sites, a 5.57 kb fragment at the 3′ side, and a MC1 promoter-driven diphtheria toxin gene (Figure 1A). Linearized pAldocVnsTV was electroporated into C57BL/6ES cells (RENKA Line) [31], and corrected targeted clones were isolated by Southern blotting. To produce germ line chimera, they were microinjected into eight cell-stage embryos of CD1 mouse strain.

Genotypes for all subsequent breeding were determined by PCR analysis of digested mouse tail samples (Figure 1B). PCR genotyping of mouse tail DNA was performed with the following primers: forward(F1), 5′- CTCTGTTAGCTGTCAAAGAG -3′; reverse 1(R1), 5′- TGGCCATGCTGCCTAGAAGC -3′; reverse 2(R2), 5′- GCTGCTAAAGCGCATGCTCC -3′. Mutated offspring were obtained from two chimera mice (230-H3 and 230-C1). Although we bred both strains, which did not show any obvious differences, only the 230-H3 strain was used in this study. There was no overt phenotype of the Aldoc-Venus heterozygous mice.

### Histological Procedures

Adult (10–14 weeks old) heterozygous Aldoc-Venus mice of both sexes were used throughout the present study unless otherwise mentioned. Adult (10–14 weeks old) wild type and homozygous Aldoc-Venus mice and young (postnatal day 5–35) heterozygous Aldoc-Venus mice were also used in some experiments. Anaesthesia with sodium pentobarbital (60 mg/kg body weight), perfusion with phosphate-buffered saline (PBS, pH = 7.4), and fixation with paraformaldehyde (4% plus 60 mM phosphate buffer, pH = 7.4) were performed and as described [32]. The dissected brain was soaked in sucrose solution (30% plus 10 mM phosphate buffer, 4°C) for two days and stored in a freezer (−80°C). To make serial section preparation, brains were coated with gelatin solution (10% gelatin, 10% sucrose in 10 mM phosphate buffer, 32°C). The chilled brain block coated in hardened gelatin was soaked for 2 nights in fixative with high sucrose content (4% paraformaldehyde, 30% sucrose in 60 mM phosphate buffer, pH 7.4). Coronal, horizontal, or sagittal sections were cut on a freezing microtome at a thickness of 40 µm and complete sets of serial sections were collected. The ventral surface of the medulla was regarded as the horizontal plane.

To obtain specimen from the retina, eyeballs were immediately dissected out from mice killed by cervical dislocation and were fixed in 4% paraformaldehyde (plus 10 mM phosphate buffer, pH = 7.4) for 3 hours, unless otherwise mentioned. The retinae were then dissected out from the eyeballs and were fixed in 4% paraformaldehyde for 16 hours. The retinal sections (50 µm) for immunostaining were cut by vibratome (VT1000S, Leica, Germany, Wetzlar).

Immunohistochemistry with fluorescent visualization of Aldoc, PLCB4, and heat-shock-protein 25 (HSP25) was performed in cerebellar sections as described [32]. Rabbit anti-Aldoc antibody (#69075, immunogen: amino acids 322–344 of rat Aldoc; produced in our laboratory [12]; 60 ng/ml), rabbit anti-PLCB4 polyclonal antibody (sc-20760, immunogen: amino acids 876–1115 of PLCβ4 of human origin, Santa Cruz Biotechnology, Santa Cruz, CA; 1∶150) and rabbit anti-HSP25 polyclonal antibody (SPA-801C, immunogen: mouse Stressgen-Gentaur, Kampenhout, Belgium; 1∶5000) were used as primary antibodies. Specificity of these primary antibodies has been confirmed by Western blot analysis (anti-Aldoc: [12]; anti-PLCB4: manufacturer’s data sheet; anti-HSP25: [33]). The same antibodies have been used in previous studies on cerebellar expression of these molecules (anti-Aldoc: [12]; anti-PLCB4: [34]; anti-HSP25: [33]). Texas Red-conjugated goat anti-rabbit IgG antibody (TI-1000, Vector Labs; 1∶333) or AlexaFluor 594-conjugated donkey anti-rabbit IgG antibody (711–585–152, Jackson ImmunoResearch; 1∶500) were used as secondary antibodies. To label nuclei in brain sections, 4′,6-diamidino-2-phenylindole (DAPI) was added to the solution of secondary antibodies (final concentration. 0.2–0.3 µg/ml).

Immunohistochemistry with fluorescent visualization was performed in retinal sections as described [35]. Rabbit anti-calbindin D-28K (AB1778, Millipore, MA, USA; 1∶1000), rabbit anti-recoverin (AB5585, Millipore, MA, USA; 1∶2000), rabbit anti-Pax6 (PRB-278P, Covance, NJ, USA; 1∶300), rabbit anti-cone arrestin (AB15282, Millipore, MA, USA; 1∶1000), mouse anti-glutamine synthetase (610517, BD Bioscience, NJ, USA, 1∶300), mouse anti-protein kinase C (PKC; 05–154, Millipore, MA, USA; 1∶5000), and chicken anti-green fluorescent protein (GFP; ab13970, Abcam; MA, USA; 1∶1000) were used as primary antibodies. These primary antibodies were visualized using Alexa Fluor 488 goat anti-chicken IgG (Invitrogen, CA, USA) and Alexa Fluor 546 goat anti-mouse and anti-rabbit IgG (Invitrogen, CA, USA). The nuclei were counterstained with 2 µg/ml of 4′,6-diamidino-2-phenylindole (DAPI; Sigma, MO, USA).

Sections were mounted on slide glass and dried. They were semi-permanently coverslipped with water-soluble embedding medium or temporarily coverslipped with PBS. Sections were photographed using a conventional fluorescent microscope (BX51WI, Olympus, Tokyo, Japan). Whole-mount specimens and sections imaged at very low magnifications were photographed using a macrozoom microscope (MVX10, Olympus). Appropriate filter sets and a cooled color CCD camera (DP-70, Olympus) were used to take photomicrographs. A confocal fluorescent microscope (LSM510, Zeiss, Oberkochen, Germany) was also used to take photographs of sections from the cerebellum. High-magnification fluorescence images of immunostained retinal sections were obtained using confocal microscope (Fv10i; Olympus, Tokyo, Japan). Photomicrographs were adjusted with regard to contrast and brightness and assembled using software (Photoshop 7, Adobe, San Jose, CA). Appropriate combination of pseudo-color was given to the fluorescent photomicrographs using Photoshop in figures that show double labeling.

After taking fluorescent photomicrographs, we stained sections of the cerebellar nucleus with thionine and permanently coverslipped with xylene-soluble embedding medium. Bright field photomicrographs were taken under a conventional microscope (BX41, Olympus) with a CCD camera (DP50, Olympus). The images were converted to gray-scale and inverted in brightness to produce fluorescence-like appearance of thionine staining. The images were then fitted and merged with the photomicrographs of Venus fluorescence to produce superimposed double staining images of Venus-thionine.

### Serial Section Alignment Analysis (SSAA)

To clarify expression patterns of Venus and other molecules in the molecular and PC layers of the cerebellar cortex from serial sections SSAA [34] was performed. The cortical layers were clipped from the low-magnification photomicrograph of coronal or horizontal serial sections in a given target area (either the rostral or caudal wall of a folium). Clipped areas of serial sections were aligned to each other with a small shift of about 100–200 µm ( = thickness of the molecular layer) to analyze continuity of the expression pattern of Aldoc. Hemispheral clipped areas were also rotated slightly to get the best alignment. SSAA was rather simple in the vermis, but not similarly simple in the hemisphere where the stripes of the Aldoc expression pattern were tilted against the parasagittal plane and the foliation of the cerebellar cortex was complicated. Therefore, we separated the hemisphere into many divisions, in which SSAA can be done locally. Results of the SSAA in neighboring areas were then combined to assemble the Aldoc expression pattern in the whole cerebellum.

### Western Blot Analysis

We used standard western blot analysis techniques, as described previously [27]. To examine Aldoc expression levels in the knock-in mice, the whole cerebellum was immediately dissected out from mice killed by decapitation under deep anesthesia with overdose of pentobarbital (50 mg/kg body weight), and was frozen in liquid nitrogen. It was later melted and homogenized in extracting buffer containing 10 mM HEPES (pH 7.4), 350 mM sucrose, 5 mM EDTA, a protease inhibitor cocktail (160–19501, Wako Pharmaceutical, Tokyo, Japan), 0.1 mg/ml benzamidine (Sigma, St Louis, MO, USA), 8 µg/ml calpain inhibitor I (Sigma) and 8 µg/ml calpain inhibitor II (Sigma). The homogenate was then centrifuged and the supernatant was stored at −80°C. The protein concentration was determined by the Lowry method. Ez apply (ATTO, Tokyo, Japan) was added to the sample. Protein samples (5 µg) were separated by electrophoresis on 12.5% sodium dodecyl sulfate-polyacrylamide gels and protein bands were then transferred from the gels to a polyvinylidene fluoride membrane (ATTO). After being blocked for 1 h with blocking solution containing 3% bovine serum albumin in Tris-buffered saline (20 mM Tris HCl, pH 8.0, 150 mM NaCl) and 0.5% Tween 20, the membrane was incubated in primary antibody against Aldoc (500 ng/ml, #69076, [12]) or in mouse anti-beta actin antibody (200 ng/ml, ab6276, Abcam, Cambridge, U.K.) overnight at 4°C in blocking solution, and was subsequently treated with goat anti-rabbit IgG peroxidase-conjugated secondary antibody (NA 935, Amersham Biosciences, Arlington Heights, IL, U.S.A.) in blocking solution. Detection was performed using chemiluminescence of 4-chloro-1-naphthol (ECL plus RPN 2132, Amersham) according to the instructions of the manufacturer. The chemiluminescence from the western blot was detected with a CCD camera (ChemiDoc XRS, Bio-Rad, Richmond, CA, U.S.A.) and optical densitometry of protein was performed using Image J (NIH, Bethesda, MD, USA).

### Labeling the Olivocerebellar Projection with a Tracer Injection

Procedures for mouse anaesthesia, surgery, and injection of tracer have been previously described [26]. We injected AlexaFluor 594-conjugated dextran amine (10,000 MW, D-22913, Invitrogen Molecular Probes, Eugene, OR, U.S.A.) solution (about 5 nl of 10% solution in saline) into the inferior olive. After seven days of survival, the mice were deeply anaesthetized and transcardially perfused. Histological procedures were the same as described above. Dextran was directly visualized by fluorescence of the conjugated dye.

## Results

### Venus Expression in the CNS of Aldoc-Venus Knock-in Mice

No differences were obvious in behavior, development, or reproduction between the homozygous (*Aldoc* ^Venus/Venus^), heterozygous (*Aldoc* ^+/Venus^) and wild-type (*Aldoc* ^+/+^) mice in ordinary breeding conditions, although detailed behavioral phenotype analysis was not performed in the present study. The size of the body and of the brain appeared similar among homozygous, heterozygous and wild type littermates (Figure 1C–E), while expression of fluorescence in the brain was observed in the heterozygote and more strongly in the homozygote (Figure 1F–H). The strain was maintained by breeding homozygous mice and heterozygous mice, which were used in the present study unless otherwise mentioned, were obtained by breeding homozygous males with normal C57BL/6N females. A total of 42 heterozygotes, four wild type mice and four homozygotes, were used in the following experiments.

**Figure 2:**
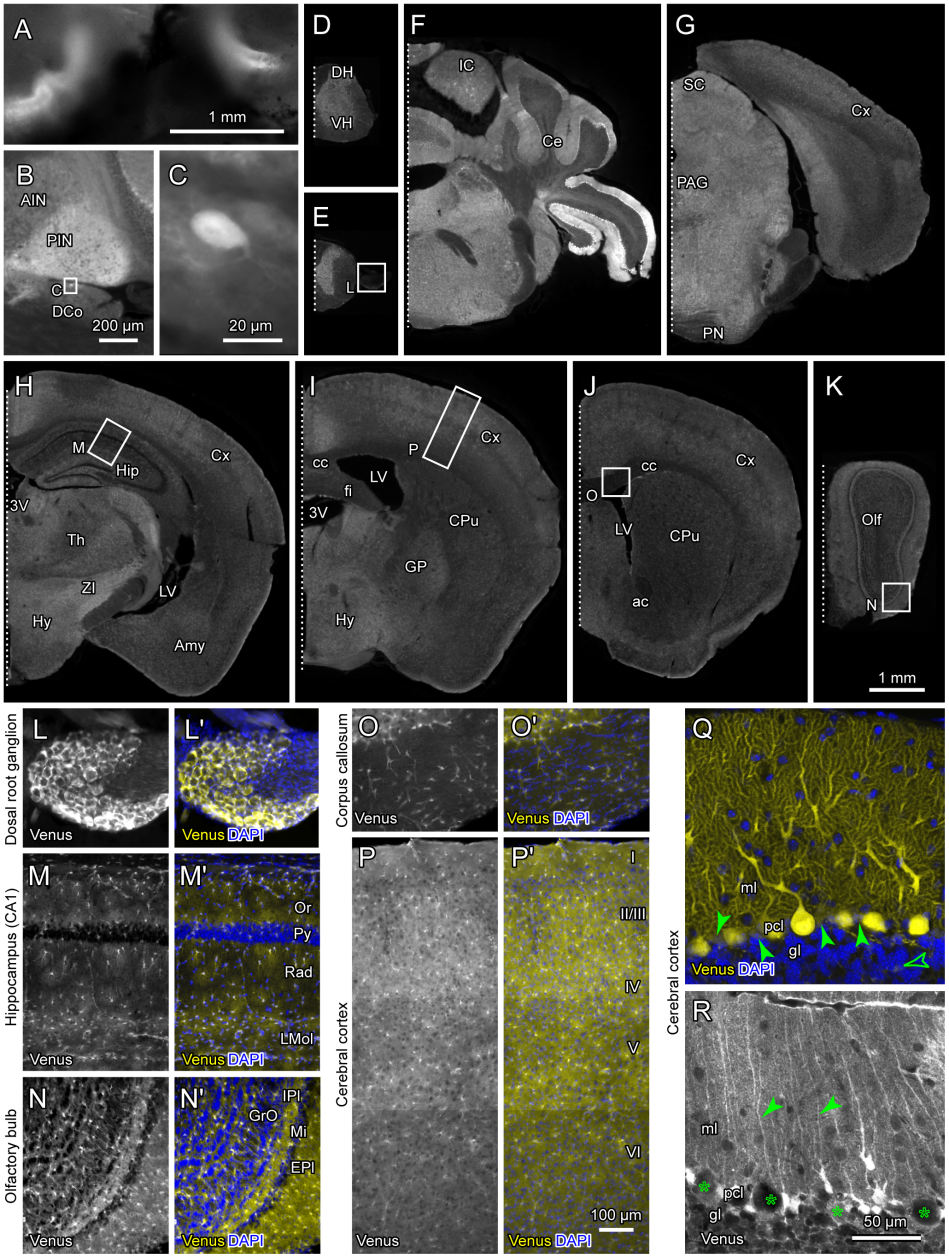
Venus expression in the nervous system other than the retina in Aldoc-Venus mice. A, Ampullas of the vertical semicircular canals. Whole mount preparation. B and C, Dorsal cochlear nucleus in a parasagittal section. High magnification (C) shows a labeled cartwheel cell. D–E, Spinal cord. F–K, coronal sections of the brain at different levels from the hindbrain to olfactory bulb. Squares (in B, E, H–K) indicate the areas that are magnified in separate panels. L–P, Double labeling with DAPI in dorsal root ganglion, CA1 of the hippocampus, olfactory bulb, corpus callosum and cerebral cortex, respectively, in medium magnification. Q, Confocal photomicrograph of double labeling of Venus and DAPI in an Aldoc-positive area of the cerebellar cortex. All PCs expressed Venus strongly at their soma, dendrites and axon terminals. Paraflocculus in a coronal section. Filled arrowheads indicate somata of Bergmann glias, while an open arrowheads indicate an astrocytes in the granular layer. Both of them expressed Venus moderately. R, Confocal photomicrograph of Venus expression in Bergmann glias in a mostly Aldoc-negative area. Lobule V in a parasagittal section. Asterisks indicate PCs. Arrowheads indicate vertical processes of Bergmann glias. Scale bar in K applies to D–K. Scale bar in P applies to L–P. Scale bar in R applies to Q and R. See the legends for Figure 1 for abbreviations.

We located the expression of Venus, which was supposed to represent Aldoc expression, throughout the CNS in heterozygotes. Since Venus had complex expression patterns in cerebellar PCs and in retinal cells, we describe the expression of Venus in these cells in separate sections. Sensory epithelium of the inner ear also expressed Venus (Figure 2A). Moderate fluorescence was observed throughout the brain and spinal cord, the intensity of which was dependent on the structure of the brain; generally gray matter had stronger fluorescence than white matter (Figures 1I–K, 2D–K). This general fluorescence reflected Venus expression in glial cells. Some parts of the vestibular nuclei also expressed fluorescence (Figure 1J), reflecting the projection of Aldoc-positive PCs. Several large round neurons with curled dendrites, which were assumed to be cartwheel cells in the dorsal cochlear nucleus, highly expressed Venus (Figure 2B, C). Cartwheel cells are akin to cerebellar PCs [36].

We looked into glial expression of Venus in several areas in detail at a higher magnification (Figure 2D–P). Double labeling using DAPI, which stains the nucleus of all cells, facilitated identifying local structures in the CNS. In the dorsal root ganglion, Venus was expressed in satellite cells (Figure 2L), which are the major type of glial cells that surround neurons in the dorsal root ganglion [37]. In the CA1 region of the hippocampus, Venus was expressed in cells that had multiple short processes and were scattered in all layers (Figure 2M, M’). Weak background Venus expression was also seen in all layers except in the pyramidal cell layer. These findings indicate that Venus was expressed in astrocytes, including fine peripheral parts of their processes that extend densely in all layers except in the pyramidal cell layer, which is filled with somata of pyramidal neurons. In the olfactory bulb, Venus positive cells were scattered in all layers, but weak background expression of Venus was dependent on the layer structure (Figure 2N, N’). In the corpus callosum, Venus was expressed in a small number of cells with fine processes, but not in the majority of cells that should represent oligodendrocytes (Figure 2O, O’). In the cerebral cortex, Venus positive cells were scattered in all layers, but weak background Venus expression was dependent on the layer structure (Figure 2P, P’). These findings indicate that Venus was expressed generally in astrocytes throughout the CNS, agreeing with the transcriptome database for astrocytes [38], and that astrocyte density varies significantly among regions.

In the cerebellum, confocal microscopy was used to identify Venus expressions in glial cells and in Purkinje cells. The complete dendritic arbor, the soma, and the entire axon are labeled with Venus in Aldoc-positive PCs (Figure 2Q, R). In addition, moderate Venus expression was also observed in the space between PCs in the PC layer. These Venus-positive spaces represent Bergmann glial cells since they often accompanied DAPI-labeled nuclei (filled arrowheads in Figure 2Q, note whitish tint of these spaces). Individual Bergmann glial cells were clearly visualized by increasing sensitivity of photography in a part of the PC layer where PCs were mostly Aldoc-negative (asterisks in Figure 2R). Processes of Bergmann glial cells were visible if the section was cut parallel to the direction of the processes, i.e., perpendicular to the surface of the cortex (arrowheads in Figure 2R). Astrocytes in the granular layer were also found to express Venus (open arrowheads in Figure 2Q). These results indicate that Bergmann glial cells and astrocytes do express Aldoc to some extent in the cerebellar cortex, although their expression of Venus was much weaker than that of typical Aldoc-positive PCs.

### Venus Expression in the Retina of Aldoc-Venus Knock-in Mice

**Figure 3:**
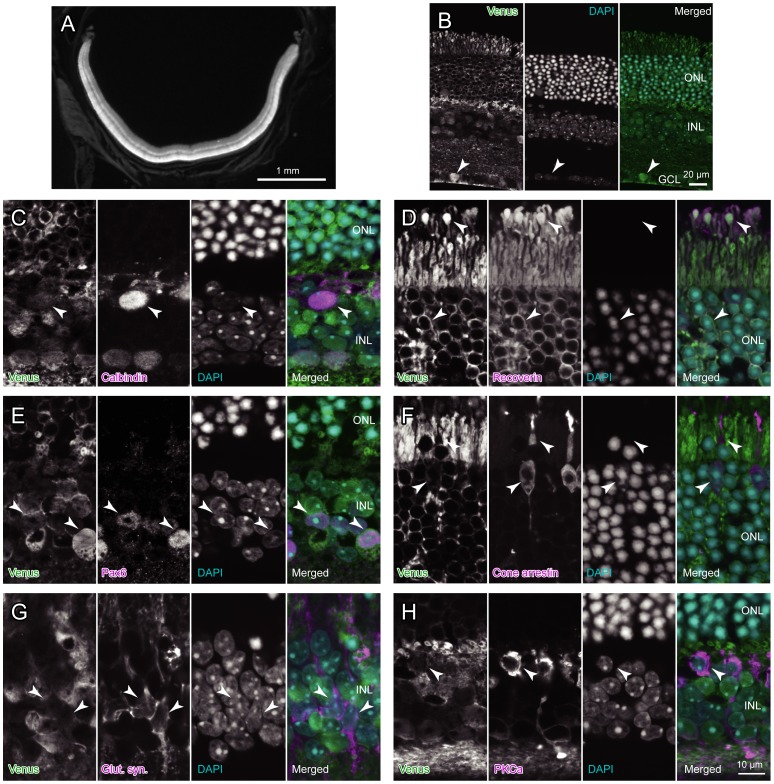
Identification of Aldoc expressing neurons and glia in the retina with confocal photomicroscopy. A, Transverse section of the retina under low magnification. Only this photo was taken with an epifluorescence microscope. This retina section was obtained from a perfused mouse. B, Intermediate magnification photos of a cross section labeled with immunostaining of Venus with anti-GFP antibody (left subpanel), DAPI staining (center subpanel) and double labeling (right subpanel). C–H, High magnification photos of cross sections labeled with immunostaining of Venus (most left subpanel), immunostaining of calbindin (C), recoverin (D), Pax6 (E), cone arrestin (F), glutamine synthetase (G) or protein kinase C (H) (next most left subpanel), DAPI staining (next most right subpanel), and triple labeling (most right subpanel). Arrowheads indicate a population of ganglion cell (B), horizontal cell (C), rod photoreceptor cells (D), amacrine cells (E), cone photoreceptor cells (F), Müller glia cells (G) and bipolar cell (H). See the legends for Figure 1 for abbreviations.

Intense expression of fluorescence was seen in the retina, so much so that the pupil of heterozygotes and homozygotes appeared greenish black. In intermediate magnification, all layers of the retina showed Venus expression (Figure 3A). The outer nuclear layer, where cone and rod photoreceptors are aligned, showed the most intense expression, while the inner nuclear layer, where bipolar, horizontal, and amacrine interneurons as well as Müller glia cells are aligned, showed the next highest expression. This agreed with a previous report [39], which showed general high Aldoc expression in the retina but not fully specified Aldoc-expressing cell types.

To determine whether all retinal cell types or only specific retinal cell types express Venus, we performed immunostaining for the marker molecules of retinal neurons and Müller glia and used confocal microscopy. Venus signal was detected highly or mildly in ganglion cells which aligned at the ganglion cell layer (Figure 3B), mildly in calbindin-positive horizontal cells which aligned at the inner nuclear layer near the outer nuclear layer (Figure 3C), highly in recoverin-positive rod photoreceptors (Figure 3D), highly or mildly in Pax6-positive amacrine cells (Figure 3E), weakly in cone arrestin-positive cone photoreceptors (Figure 3F), weakly in glutamine synthetase-positive Müller glia cells (Figure 3G), and mildly in protein kinase C-positive bipolar cells (Figure 3H). Although the Venus fluorescent signals were weaker in cone photoreceptors and Müller glia cells, these results suggest that all retinal cell types, which were identified with the marker molecules, express Venus.

### Venus Expression Accurately Recapitulated Aldoc Expression in Cerebellar PCs

To compare the fluorescence with the Aldoc expression, we measured Aldoc expression level in the cerebellum with Western blot (Figure S1G). The Aldoc expression level in the heterozygote was about half (47.1%) of that in the wild-type and the homozygote showed no expression of Aldoc (Figure S1H). These results indicated that the Aldoc expression was partially and completely replaced by Venus expression in the heterozygote and in the homozygote, respectively. Specificity of the anti-Aldoc antibody, which was produced in our laboratory against the rat amino acid sequence [12], was also confirmed in mouse cerebellar tissue with the Western blot.

The Venus expression was then compared with Aldoc expression in the cerebellar sections. Concordant with the results of Western blot, Aldoc expression was seen in multiple stripe-shaped distributions of PC subsets in the wild-type and with a lower intensity in the heterozygote (Figure S1D–F). On the contrary, the cerebellar stripe-shaped fluorescence expression was absent in the wild-type, while moderately present in the heterozygote and strongly present in the homozygote (Figure S1A–C).

**Figure 4:**
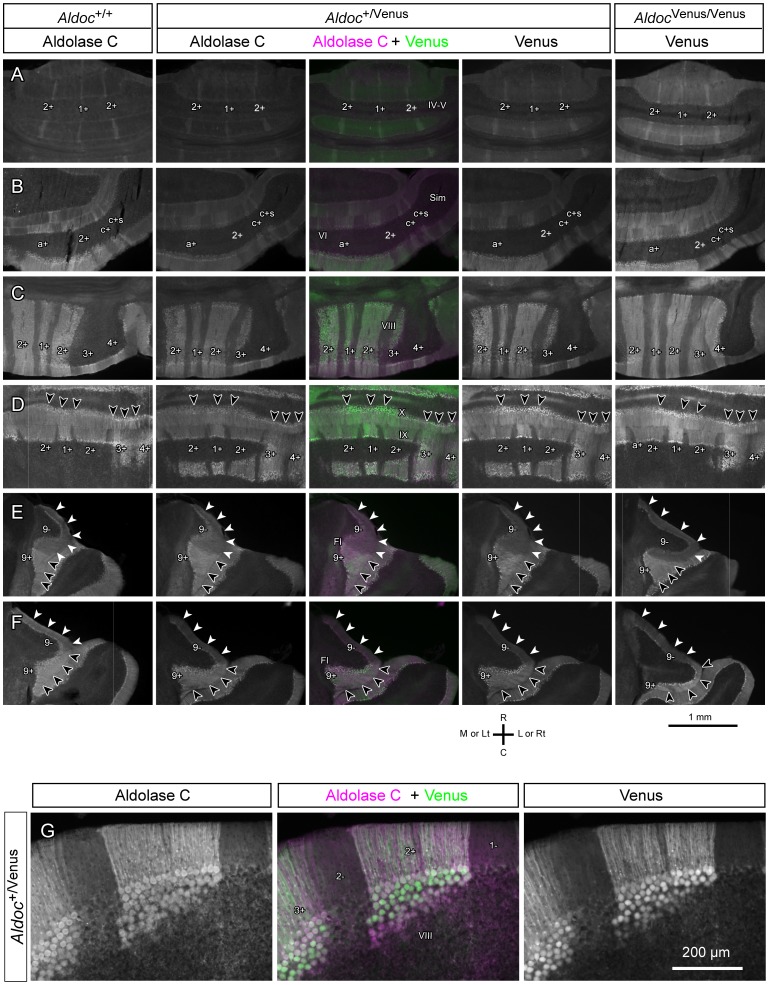
Comparison of the striped Aldoc or Venus expression pattern among wild type (*Aldoc* ^+/+^), heterozygote (*Aldoc* ^+/Venus^) and homozygote (*Aldoc* ^Venus/Venus^) mice. A–F, Photomicrographs of Aldoc immunostaining in a wild type (the most left column), Aldoc immunostaining, double labeling and Venus in a heterozygote (the three center columns), and Venus in a homozygote (the most right column) in horizontal sections of the cerebella at the similar levels (A, rostral cerebellum with three narrow zones; B, Paravermal lobule VIa; C, Lobule VIII; D, Junction between lobules IXc and X; E, Flocculus, dorsal part; F, Flocculus, ventral part). Arrowheads in D indicate areas with higher Aldoc/Venus expression in lobule X. White and black arrowheads in E and F indicate rostral and caudal parts of the flocculus that had different Aldoc/Venus expression levels. Sections from the wild type and heterozygote were processed with Aldoc immunostaining with Texas red-conjugated secondary antibody in the same session with the same antibody solutions. Photos of Aldoc immunostaining and Venus were, respectively, processed with the same exposure and adjustment settings for all mice in A–F. Scale bar under F applies to A–F. G, Higher magnification photomicrographs of Aldoc immunostaining (left panel), double labeling (center panel) and Venus (right panel) of a coronal section of the cerebellum (lobule VIII) in a heterozygote, showing complete match at the cell population level.

We then compared the striped Aldoc expression pattern in the cerebellum among the wild type and heterozygous and homozygous mutants to confirm that the Venus expression pattern in the mutants represent the Aldoc expression pattern of the wild type mouse. The striped patterns of Aldoc and/or Venus expression were compared in serial horizontal sections of these mice (Figure 4). Note that the Aldoc protein expression was weaker in the heterozygote than in the wild type and that Venus expression was weaker in the heterozygote than in the homozygote. Besides this intensity difference, nearly the same spatial conformation of the Aldoc/Venus expression pattern was observed in any area of the cerebellum among the wild type and heterozygous and homozygous mutants (Figure 4A–F). Differences between the wild type and mutant cases were slight within the range of random inter-individual variation (cf. section “Little inter-individual variation in the striped Aldoc expression pattern in the cerebellar cortex”). In other experiments in which cerebella were cut in serial coronal sections, the striped pattern of Aldoc/Venus expression in heterozygous and homozygous mutants were also virtually the same as that in a wild type (not shown). Thus, it was confirmed that the Venus expression pattern in Aldoc-Venus mutant mice exactly reflected the Aldoc expression pattern of the wild type mice.

In the heterozygote, in which both Aldoc and Venus are expressed, the Aldoc expression pattern and the fluorescence expression pattern exactly coincided with each other (Figure 4A–F, three center columns). At high magnification, expression of Aldoc and Venus were seen exactly in the same subsets of PCs and in the same populations of glial cells, presumably astrocytes, in the cerebellar cortex of the heterozygote (Figure 4G). The expression intensity of Aldoc in PCs was not simply dichotomous into positive and negative subsets, but instead graded variation was seen in some areas. Such intensity variation in the Aldoc expression was generally well recapitulated by intensity variation in the Venus expression (Figure 4G), although the Aldoc and Venus labelings were not completely parallel with each other as seen by variable green/magenta tint in the merged photo (Figure 4G, center). We think this small disagreement may be explained by technical issues such as (1) weaker immunostaining in the center of section thickness and (2) possible different intracellular distribution of Aldoc and Venus proteins. As a whole the results indicate that the striped Venus expression pattern in this mouse strain can be regarded as faithfully representing the intrinsic Aldoc expression pattern of the wild-type mouse in the cerebellum. In the present study, we used heterozygotes of this mouse strain (*Aldoc* ^+/Venus^) to examine the detailed intrinsic Aldoc expression pattern in the cerebellum by using its intrinsic Venus expression.

### Development of the Striped Aldoc Expression Pattern in the Cerebellum

**Figure 5:**
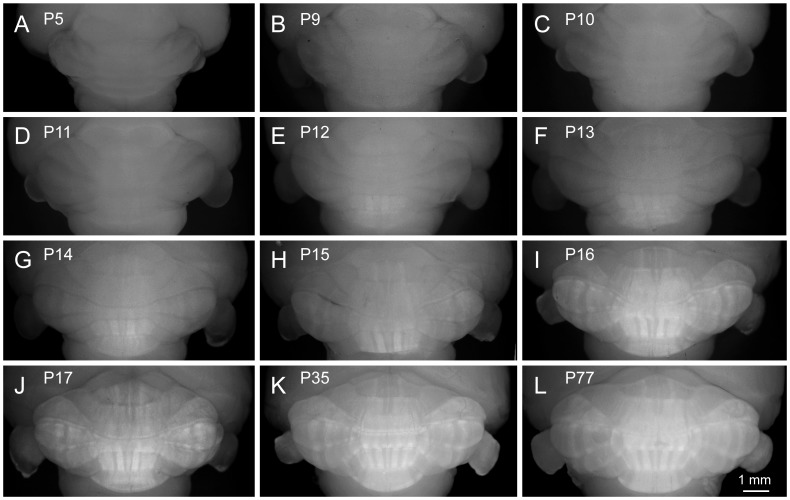
Development of Venus expression pattern in the cerebellum. A–L, Photomicrographs of dorsocaudal aspect of the cerebellum in whole-brain preparations of heterozygotes at P5, every postnatal day between P9 and P17, P35 and P77. All photomicrographs were taken and adjusted with the same protocol. Scale bar in L applies to A–L.

We compared cerebellar Venus expression in the fixed whole mount preparation of heterozygotes at every postnatal day between P5 and P17, and at P35 and P77. Although we saw some inter-individual variation in Venus expression during development, representative samples are shown for different postnatal days in Figure 5. Between P5 and P8, there was moderate Venus expression throughout the brain (Figure 5A). Between P9 and P11, the general Venus expression became gradually stronger in the cerebellum and a faint striped pattern of expression gradually emerged in lobule VIII and IX (Figure 5B–D). At P12, the striped expression of Venus appeared nearly throughout the entire cerebellar cortex, though faintly except in lobules VIII and IX (Figure 5E). This striped expression pattern of Venus in the cerebellar cortex became gradually more intense between P12 and P17 (Figure 5E–J). The cerebellar cortex at P17 expressed Venus in the striped pattern nearly as clearly as in the adult (Figure 5J). After P17, individual stripes became clearer although intensity of Venus expression did not much increase (P35 and P77, Figure 5K, L). The striped pattern that emerged in the developmental stages was nearly identical to the striped pattern in adult. The developmental emergence of the striped Venus expression in the cerebellum was basically similar to the development of striped Aldoc expression reported in the cerebellar cortex of the mouse [40] and of the rat [41]. However, concerning the general expression level of the protein in the cerebellum, transient high expression of Aldoc (zebrin II) at P12–P15 in mouse and rat [40], [41] was not obvious in Venus expression observed through epifluorescence of the cerebellar whole mount preparation.

### Aldoc Expression Pattern in the Vermis and Hemisphere

**Figure 6:**
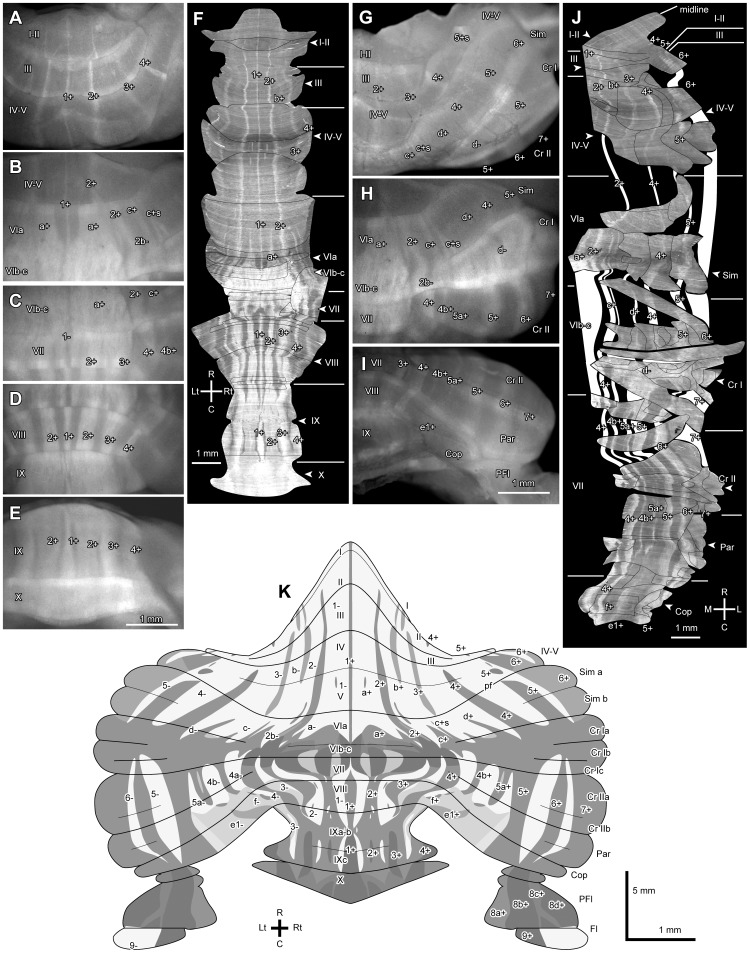
Detailed Aldoc expression pattern in the cerebellar vermis and hemisphere. examined in Aldoc-Venus mice. A–E, Aspects of vermal surface. F, Reconstruction of the unfolded entire vermal cortex (molecular layer) by SSAA from serial sections. The rostral and caudal parts (lobules I–V and lobules VII–X) were from serial coronal sections in a mouse and the central part (lobule V–VII) were from serial horizontal sections in another mouse. G–I, Aspects of hemispheral surface. J, Reconstruction of the unfolded entire hemispheral cortex (molecular layer) by SSAA from serial sections. Since folial organization is complicated, SSAAs in individual folia are combined to show the entire cortex as a mosaic of the SSAAs. K, The revised scheme of the Aldoc expression pattern drawn on the unfolded cerebellar cortex based on the SSAAs shown in F and J. Note that darker colors mean more intense expression of Venus in K, opposite to photomicrographs. The mosaic displays of SSAAs were shrunk by 45% in the rostrocaudal (vertical) direction to fit to the space in F and J. Arrowheads indicate the apex of lobules in F and J. Scale bar in E applies to A–E. Scale bar in I applies to G–I. See the legends for Figure 1 for abbreviations.

In the remainder of the present study, we analyzed detailed Aldoc expression patterns in the cerebellum by taking advantage of the fact that Venus expression exactly represents Aldoc expression in the Aldoc-Venus mouse. We cut serial coronal, sagittal, and horizontal sections of the entire cerebellum of heterozygotes (n = 6) and photographed every section. We then performed serial section alignment analysis (SSAA) [34] to trace the spatial expression pattern of Venus throughout the cerebellar cortex in detail. The final summary of SSAA for the entire cerebellar cortex (Figure 6F, J) was obtained from sections from three brains; serial horizontal sections were used in lobules VI–VII, serial coronal sections were used in lobules I–V and VIII–X, and serial parasagittal sections were used in the paraflocculus and flocculus. The results of this SSAA were compared to the photomicrographs of different aspects of the cerebellar surface of the Aldoc-Venus mouse to confirm identity of the stripes (Figure 6A–E and G–I). Based on these results, we revised (Figure 6K) our previous scheme for Aldoc expression pattern, which was produced based on Aldoc immunostaining of the cerebellar cortex in the ICR mice [26]. The revised scheme showed finer differences in expression intensity than did the previous scheme. In addition to being able to confirm major stripes that had been previously described, we could also recognize several detailed striped patterns that were not clearly described before, including satellite stripes and intensity changes within a stripe as seen in lobule VII-X in the vermis. In particular, we were able to recognize an Aldoc-negative area in the flocculus. Here, we mainly focus on these newly described patterns.

In the vermis, the median Aldoc-positive stripe (1+) was seen in all lobules except in rostral lobules VII. The paramedian positive stripe a+ appeared in lobule VIa, widened greatly in caudal lobule VIa, lobule VIb–c and rostral lobule VII, where the same stripe is designated as 2+, narrowed in caudal lobule VII and lobule VIII, and continued throughout lobule IX. Several satellite stripes of negative or faint Aldoc expression were seen within 2+ in lobules VII, VIII and IXa–b. The next paramedian stripe (2+ in lobules II–VI and 3+ in lobules VII–IX) appeared in lobule II and disappeared in the rostral folial wall of lobule IXa–b (Figure 6F, K). In lobule IXc, 1+ and central part of 3+ and lateral part of 4+ had higher expression than other parts of positive stripes. These sub-stripes of higher expression continued and became wider in lobule X (Figure 6F, K).

In the lateral vermis, stripes b+ and 3+ were seen lateral to stripe 2+, as was reported previously in lobules I–V [26]. In lobule VIa, the midlateral part of 2+ expressed Venus less intensely than the wide medial and narrow-most lateral part of 2+ (Figure 6F). It was not straightforward in the present analysis to reconfirm 2b+ as defined previously in the ICR mouse [26]. However, careful comparison between the specimens in the present study and those from our previous study [26] indicated that the previously defined stripe 2b+ was equivalent to the narrow most lateral part of 2+ in lobule VIa (Figure 1D, E, F of [26]), which sometimes appeared to be laterally separated from the main part of 2+ (asterisks in Figures S2L, S3O, S4H). The negative stripe immediately lateral to 2+ or 2b+ (designated as 2b− as in [26]) was most extended caudally from lobule VIa towards the apex of lobule VIb–c as observed previously (Figure 4 of [26]). In the transition area between the vermis and pars intermedia in lobule VI-simple lobule, c+ was consistently observed lateral to stripe 2+ in all mice together with a further lateral narrow satellite stripe (designated “c+s”) separated by a narrow gap between c+ and c+s (Figure 6B, F, H, K). The spatial arrangement of stripes 2+, 2b−, c+ and c+s made characteristic appearances in the lateral lobule VIa and the medial simple lobule, a landmark for identifying stripes in this area. In the lateral vermis in lobule VIb–c, all positive stripes merged. In lobules VII–VIII, a positive stripe 4+ was located lateral to 3+.

In the caudal folial wall of lobule VIII and in the rostral folial wall of IXa, 3+ and 4+ were clearly distinguished. Stripe 4+ in lobules VII and VIII was continuous exclusively to stripe 3+ in lobule IXa–b (Figure 6F, K).

In the hemisphere, SSAA was done separately for individual lobules or for parts of individual lobules, due to complicated curvature of the folia. The results of SSAA were then matched spatially with each other, by carefully tracing identifying stripes, to show the continuity of stripes in the whole hemispheral cerebellar cortex to the greatest extent possible (Figure 6J). In the rostral hemisphere (lobule II-crus I), stripe 4+ appeared in lobule IV and continued to crus I, where it merged with other positive stripes. A small positive area was located at the position equivalent to stripe 4+ in the rostral edge of hemispheral lobule II–III (also designated 4+, Figures 6K, S3P, S5N). Stripe 5+ appeared in the bottom of the fissure between lobule V and the simple lobule and continued to crus I, where it merged with other positive stripes. Three separate and small positive stripes were located at the position equivalent to stripe 5+ in hemispheral lobules IV–V and II–III (also designated 5+, Figures 6K, S3Q, S5N). Stripe 6+ appeared in lobule II–III, split in medial and lateral parts in lobules IV–V, and continued to crus I. In the rostral pars intermedia, stripe d+ was sometimes difficult to recognize in the surface of the simple lobule since it was not very intensely labeled. However, SSAA clearly located d+ between c+s and 4+ in the simple lobule and in rostral crus I (Figure 6J, K). The negative stripe between d+ and 4+, i.e. d−, entered very caudally toward the apex of crus I (Figure 6H, J).

In the caudal pars intermedia, 5a+ appeared to be divided into two stripes since the center of 5a+ was less intense than its medial and lateral parts in crus II (Figure 6J). Stripes 4b+ and 5a+ were traced from crus I to the paramedian lobule, where they became wide and disappeared. Stripe 4b+ reappeared for a short distance near the bottom of the fissure between the paramedian lobule and copula pyramidis. In the copula pyramidis, mildly positive stripes f+ and e1+ were located as reported previously [26]. Stripe e2+, which was located in the lateral part of e+ in the rat [12], could be hardly recognized. In the caudal hemisphere, stripes 5+, 6+ and 7+ was observed as reported before [26].

All of these stripe patterns were mapped in the unfolded scheme of the cerebellar cortex (Figure 6K). As summarized in the above, the present analysis with the Aldoc-Venus mouse showed several detailed characteristics that had not been clearly reported so far in the striped Aldoc expression pattern. When we looked into the Aldoc immunostaining in the wild type, indeed, all these characteristics could also be recognized in the wild type (for example, satellite stripe c+s in lateral lobule VIa, Figure 4B; intensity differences within Aldoc positive areas in lobule X, arrowheads in Figure 4D).

### Little Inter-individual Variation in the Striped Aldoc Expression Pattern in the Cerebellar Cortex

**Figure 7:**
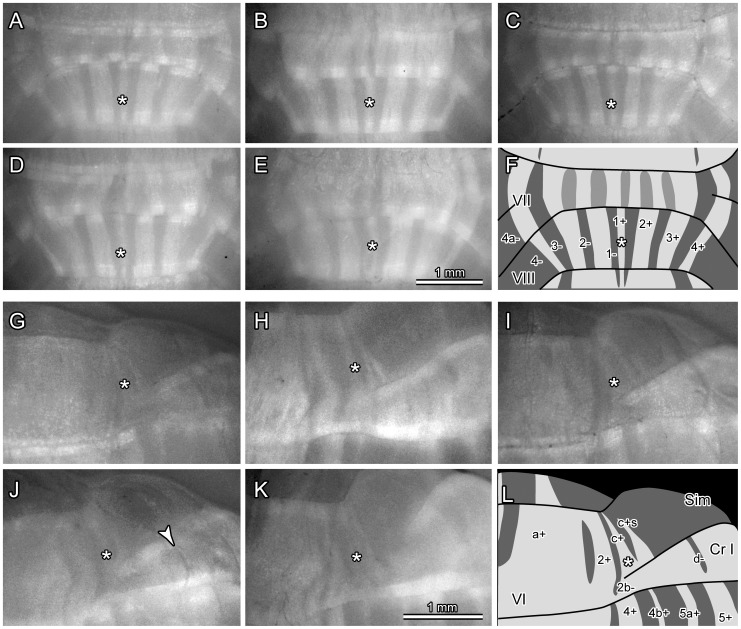
Inter-individual variation in Venus expression pattern in the cerebellum. A–F, Photomicrographs of vermal lobules VII–VIII in five adult mice (A–E) and a schematic representation of the striped expression pattern of Venus in this area (F). G–L, Photomicrographs of the right side of lobules VI and its hemispheral extension, simple lobule and crus I in five adult mice (G–K) and a schematic representation of the striped expression pattern of Venus in this area (L). For abbreviations of anatomical terms, see the legends for Figure 1. Arrowhead in J indicates a trace of the blood vessel. Scale bar in E applies to A–F. Scale bar in K applies to G–L.

It was reported that major Aldoc stripes have little inter-individual variation in the rat [22]. Since inter-individual variation was examined simply by looking at the striped pattern in the outer aspects of the whole cerebellar preparation in Aldoc-Venus mice, we re-examined it in two particular areas of the cerebellum: vermal lobules VII and VIII (Figure 7A–F) and in lateral vermal lobule VI (Figure 7G–L). The striped pattern is particularly clear in lobule VIII, in which the midline stripe (1+, asterisks in Figure 7A–E) is narrower than the second and third lateral stripes, as represented in the scheme. The pattern was consistent for all examined individuals with little inter-individual variation (Figure 7A–E).

In lateral vermal lobule VI, we showed that the spatial arrangement of stripes 2+, 2b−, c+ and c+s made characteristic appearances in lateral lobule VIa and in the medial simple lobule (preceding section). In the sample photos of this area for five individuals, a narrower positive stripe (2+) was located lateral to the wider paramedian stripe (a+). Lateral to 2+, a negative stripe (2b−) extended down toward the apex of the transitional area between lobule VIb–c-crus I and, lateral to this 2b− stripe, a narrower positive stripe (c+, asterisk in Figure 7G–K) was located. Then, lateral to it, separated by a very narrow negative stripe, a narrow positive stripe (c+s) was seen. This pattern was consistently seen in each of the five individuals, with the stripe c+s divided mediolaterally into two stripes in two cases (Figure 7H, I). These results indicate that the striped expression pattern of Aldoc was generally consistent among individuals with minor inter-individual variation, even concerning narrow stripes that were identified for the first time in the present study.

Dark narrow curved lines that were seen in variable places on the cerebellar surface (Figure 7J, arrowhead) were the trace of blood vessels and were not considered as Venus stripes in the above analysis.

### Aldoc Expression Pattern in the Paraflocculus and Flocculus

The paraflocculus and the flocculus have been generally thought to be Aldoc-positive in the rat [12], [42] and in mice [26]. Although some patterns were previously observed, these patterns were not clearly identified because the three-dimensional structure of these lobules is complicated [26] and these areas somehow had higher non-specific background labeling than other areas in immunostaining with DAB visualization (see Discussion).

**Figure 8:**
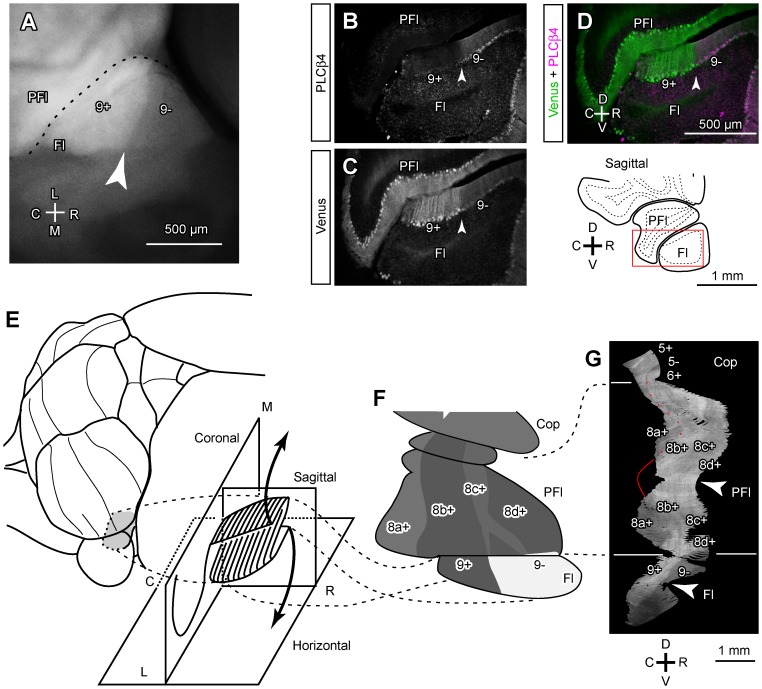
Venus expression pattern in the flocculus and paraflocculus. A, Photomicrograph of the flocculus in the ventral outer surface of the whole-mount preparation of the right cerebellum. Venus expression in the rostral part was weaker than in the caudal part. B–D, Photomicrograph of double labeling of PLCB4 in a parasagittal section of the flocculus. Superimposing (D) of immunostaining of PLCB4 (B) and Venus fluorescence (C) in the same section indicate that PLCB4 and Venus are expressed in a complementary pattern; the rostral part that was weak in Venus expression expressed PLCB4. Arrowheads indicate the boundary between the Venus-positive and -negative areas. Inset in the right bottom indicates the position of the photomicrograph. E, Schematic illustrating lobular organization and unfolding of the flocculus. F, The Aldoc expression pattern in the paraflocculus and flocculus depicted in the unfolded scheme, based on the SSAA (G). G, SSAA from serial parasagittal sections of the paraflocculus and flocculus. The displays of SSAA were shrunk by 50% in the dorsoventral (vertical) direction. Red curve indicate the boundary between stripes 8a+ and 8b+. Arrowheads indicate the apex of lobules. Scale bar in D applies to B–D. See the legends for Figure 1 for abbreviations.

In whole-mount preparations of Aldoc-Venus mice, we readily noticed that the rostral part of the flocculus had lower Venus expression than its caudal part (Figure 8A). Therefore, we looked into the Aldoc/Venus expression pattern in the flocculus and neighboring paraflocculus.

We schematically unfolded these lobules and added them to the unfolded scheme of the whole cerebellar cortex in order to map Venus expression patterns from SSAA (Figure 8E–G). Note that the general orientation of the cortex is rotated in these lobules; the caudal and rostral directions in the paraflocculus and flocculus are equivalent to the medial and lateral directions in other areas of the cerebellum in the mouse due to developmental rotation of these lobules [34].

In the present study, SSAA from serial parasagittal sections in Aldoc-Venus mice clearly revealed longitudinal striped patterns in these lobules. In the dorsal part of the paraflocculus, four longitudinal stripes; i.e. less intense, intense, less intense, intense expression intensities were observed from the caudal to rostral part (tentatively named stripes 8a+, 8b+, 8c+, 8d+; Figure 8F, G). These stripes were continuous to the ventral part of the paraflocculus.

In the flocculus, a clear striped pattern of Venus expression was observed in SSAA; expression was stronger in the caudal part than in the rostral part (tentatively named 9+ and 9−, respectively) (Figure 8F, G). Since the expression level of Venus in the rostral part (9−) was similar to that of other Aldoc-negative areas in the cerebellar cortex (Figure 4A–D vs. E, F), we regard this area as Aldoc-negative in the present study. Although the Aldoc-negative stripe in the rostral flocculus has not been reported in the rodent so far, we could recognize this negative stripe not only in the heterozygote but also in the wild type when we looked into Aldoc immunostaining (white arrowheads vs. black arrowheads in the most left column of Figure 4E, F). There was no obvious difference in the striped pattern (9+ and 9−) between the wild type and the heterozygote.

In the whole-mount preparation, no stripes were clearly recognized in the paraflocculus (not shown). It was likely that the stripes were less clear in the whole-mount preparation than in the SSAA of serial sections presumably because (1) scattering of fluorescence by the tissue causes general blurring, (2) fluorescence comes not only from the folial apex that faces the cerebellar surface but also from the folial wall, and (3) vasculature in the cortex or in the surface produces some “noise” in fluorescence. Thus, it was not unreasonable that the stripes that were observed in SSAA were difficult to recognize in the surface of the paraflocculus in the whole-mount preparation. However, in the flocculus, a boundary between the rostral Aldoc-negative part and the caudal Aldoc-positive part was clearly recognized in the whole-mount preparation (Figure 8A). To confirm the Aldoc-negative area in the rostral flocculus, we examined the expression of phospholipase Cβ4 (PLCB4), which is expressed specifically in Aldoc-negative areas in the cerebellar cortex [19], and found that the rostral part of the flocculus expressed PLCB4 (Figure 8B). The boundary between the PLCB4-positive and -negative areas, and that between the Venus-negative and -positive areas, coincided with each other (Figure 8B–D). The result supported the conclusion that the flocculus is separated into a rostral, Aldoc-negative (and PLCB4-positive) part, and a caudal, Aldoc-positive (and PLCB4-negative) part.

### Relationship between Aldoc Stripes and Functional Zones in the Flocculus Analyzed by Double Labeling with HSP25 and Olivocerebellar Projection

Regarding its involvement in controlling different types of eye movements, the flocculus is divided into three major, and some minor, functional zones in the mouse [43] and in other mammals [44], [45]. Basically, the central zone (vertical axis zone, zone 2) receives innervation from the dorsal cap subnucleus of the inferior olive and is mainly involved in horizontal eye movements, whereas the caudal and rostral zones (horizontal axis zones, zones 1 and 3) receive innervation from the ventrolateral outgrowth subnucleus of the inferior olive and are mainly involved in vertical eye movements [46]. Additional smaller zones have also been recognized (zones C2, 0 and 4). These functional zones of the mouse flocculus are marked by topographic projections from the inferior olive (above), and also by HSP25, which is expressed in subsets of PCs that overlap PCs in most of zone 1 and in a small caudomedial part of dorsal zone 2 [43].

**Figure 9:**
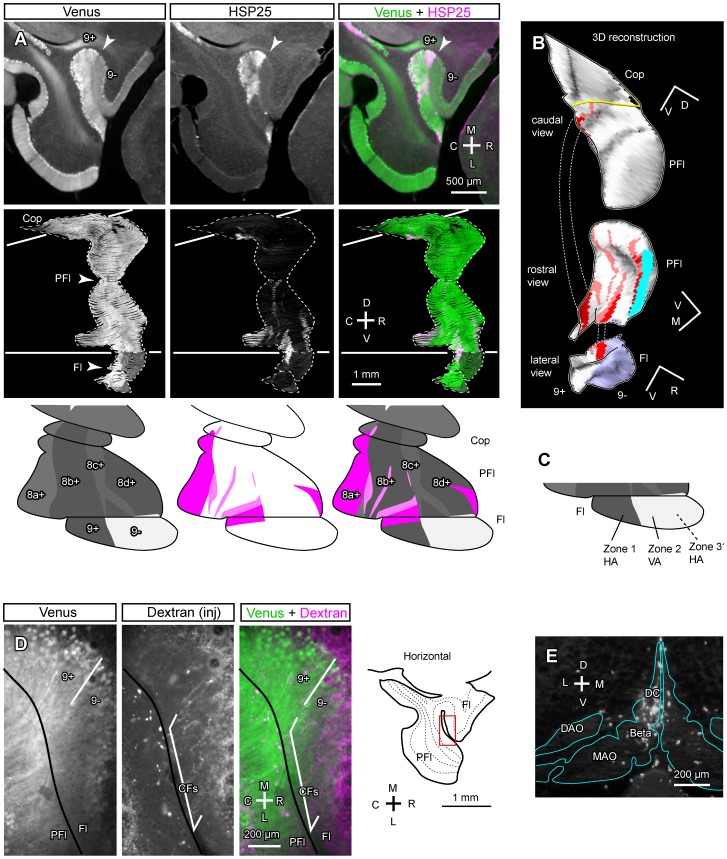
Relationship of the 9+/9− boundary in the flocculus with the known floccular compartmentalization. A, Comparison of the Venus expression pattern (left), the immunostained HSP25 expression pattern (center) and double labeling (right) in photomicrographs of a horizontal section (top), SSAA from serial horizontal sections (middle), and mapping in the unfolded scheme (bottom), of the paraflocculus and flocculus. Arrowheads in the top panels indicate the boundary between Aldoc-positive and -negative areas, which coincided with the boundary between HSP25-positive and -negative areas, in the flocculus. Arrowheads in the center panels indicate the apex of the lobule. The displays of SSAA were shrunk by 50% in the dorsoventral (vertical) direction. The mapping was based on SSAA. B, Three dimensional reconstruction of the HSP25 and Venus labeling patterns in the paraflocculus and flocculus. Caudal and rostral views of the paraflocculus and a lateral view of the flocculus are shown. Red and pink areas indicate strong and moderate HSP25 expression. Purple areas indicate Aldoc-negative area. C, Schematic showing correspondence between the Aldoc stripes and functional zones in the flocculus. D, Anterograde labeling of olivocerebellar climbing fibers by a tracer injection into the dorsal cap subnucleus of the inferior olive. Comparison of labeled climbing fibers (center), Venus expression (left) and double labeling (right) indicates that labeled climbing fibers were located in the Aldoc-negative area. E, Injection site of tracer (Alexa Fluor 594-conjugated dextran amine) in the dorsal cap subnucleus of the inferior olive. Contour of the inferior olive subnuclei (blue) was drawn by referring to another photo of intrinsic and Venus fluorescence of the same section. See the legends for Figure 1 for abbreviations.

Here we attempted to clarify the relationship between the newly found Aldoc stripes and the functional zones of the mouse flocculus by studying the HSP25 expression pattern as well as the olivary projections in the Aldoc-Venus mouse. In the flocculus and the paraflocculus, HSP25 was expressed in multiple longitudinal stripes as reported previously. In the medial flocculus, the HSP25 positive area almost overlapped the most dorsal part of the Aldoc-positive stripe 9+ (arrowheads in Figure 9A top panels). The combination of SSAA of double-labeled serial horizontal sections of the paraflocculus and the flocculus (Figure 9A) and the three dimensional reconstruction of the HSP25 expression pattern in the paraflocculus and the flocculus (Figure 9B) revealed a detailed striped pattern of HSP25 expression in these areas. These results were summarized in the unfolded scheme of the flocculus and paraflocculus (Figure 9A bottom panels). The largest HSP25-positive area in the paraflocculus was found to be located in stripe 8a+. The largest HSP25-positive area in the flocculus overlapped the most dorsal part of Aldoc-positive stripe 9+ (Figure 9A middle panels). Since the largest HSP-25-positive area in the flocculus corresponds to zone 1 [43], these results indicate that the Aldoc-positive stripe (9+) corresponds to zone 1, while the Aldoc-negative stripe 9− covers zone 2 (Figure 9C). To check this correspondence, we labeled the olivocerebellar projections. Climbing fibers labeled by fluorescent tracer injection into the dorsal cap (Figure 9E) were observed in Aldoc-negative stripe 9−, but not in Aldoc-positive stripe 9+ (Figure 9D). Since the dorsal cap projects to zone 2 [44], [45], these results support our conclusion that Aldoc-negative stripe 9− covers zone 2.

SSAA of the double-labeled sections also allowed us to reconfirm the multiple longitudinal stripes of HSP25 expression in lobules VI–VII and IX–X [33] (Figure S6). The positional relationship between the HSP25 stripes and the Aldoc stripes was summarized in the map of Aldoc stripes drawn on the unfolded scheme of the cerebellar cortex (Figure S6J).

### Cerebellar Nuclei

**Figure 10:**
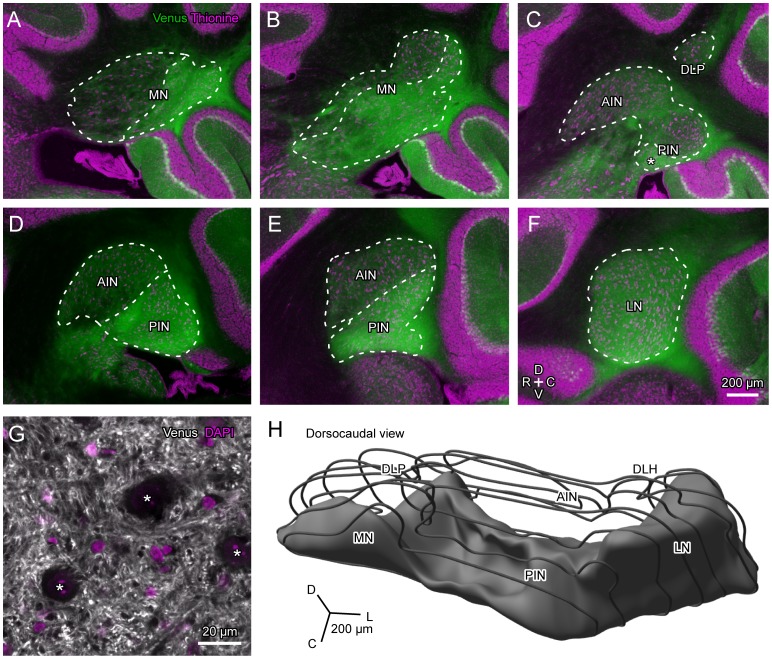
Venus expression pattern in the mouse cerebellar nuclei. A–F, Parasagittal sections showing the cerebellar nuclei at different mediolateral levels, separated by about 0.24 mm. After taking photos of Venus fluorescence (green), the sections were stained with thionine (magenta) and photographed again. Two photos were superimposed to produce double-stained images (see Methods). Thus, the photos can be regarded as double labeling of Aldoc and thionine. Dotted curves indicate contours of the cerebellar nuclei and boundaries of Aldoc-positive and -negative divisions of the cerebellar nuclei. Asterisk in C indicates the caudoventral crust-like aldolase C-positive area in the posterior interposed nucleus. G, Confocal photomicrograph of the Aldoc-positive part. Asterisks in G indicate large neurons in the cerebellar nucleus. H, Three dimensional reconstruction of the right cerebellar nuclei of mouse. While the contour of the entire cerebellar nuclei is shown with wire frames, the Aldoc positive part is shown with gray solid. Scale bar in F applies to A–F. See the legends for Figure 1 for abbreviations.

Cerebellar nuclei have previously been subdivided into the rostrodorsal Aldoc-negative part and the caudoventral Aldoc-positive part in the rat [47]. These subdivisions have been determined by the projection pattern of Aldoc-positive and -negative PCs since neurons in the cerebellar nuclei do not appear to express Aldoc. Indeed, confocal microscopy showed that axons and axonal terminals express Venus, but the somata of nuclear neurons lacked Venus expression in the Aldoc-positive areas of the cerebellar nucleus in the Aldoc-Venus mouse (asterisks in Figure 10G). We examined whether a similar division of Aldoc-positive and -negative areas was present in the cerebellar nuclei in the Aldoc-Venus mouse.

Thionine staining of the sections allowed us to depict contours of the cerebellar nuclei, as well as the boundaries between the positive and negative areas, in individual sections. Generally, the boundary between the Aldoc-positive and -negative parts was simple. In the medial nucleus, Venus expression was low (Aldoc-negative) in the rostrodorsal part but high (Aldoc-positive) in the caudoventral parts (Figure 10A). The dorsolateral protuberance of the medial nucleus was Aldoc-negative (Figure 10C). The anterior interposed nucleus was entirely Aldoc-negative (Figure 10C–E). The posterior interposed nucleus had a complex expression pattern of Aldoc. In the medial part of the interposed nucleus, the Aldoc-negative area occupied most of the posterior interposed nucleus, except for its most ventral crust-like area (Asterisk in Figure 10C). On the other hand, the lateral part of the interposed nucleus was entirely Aldoc-positive. The lateral nucleus was completely Aldoc-positive (Figure 10F). These expression patterns in the mouse cerebellar nuclei generally resembled the Aldoc expression pattern in the rat cerebellar nuclei [47]; the cerebellar nuclei were divided into the caudoventral Aldoc-positive and rostrodorsal Aldoc-negative parts. This is shown in the three-dimensional reconstruction of the mouse right cerebellar nuclei, in which the gray solid represents the Aldoc-positive part (Figure 10H).

## Discussion

The expression pattern of Aldoc (zebrin II) has previously been studied in the cerebellum, retina and other brain areas through immunostaining [11], [12], [22], [39]. Here, we undertook a more systematic and detailed study of Aldoc expression patterns than those in previous studies in response to the increasing interest in the functional property of this molecule, by generating a new mouse strain (the Aldoc-Venus mouse) in which Aldoc expression is visualized through the innate expression of a mutated green fluorescent protein. Since the Venus gene is inserted into the genome by knock-in mutation in this strain, Venus gene expression can generally be assumed to represent the *Aldoc* gene expression pattern accurately [48]. Indeed, there was no detectable difference in the striped pattern of Aldoc or Venus expression in the cerebellar cortex between the wild type and Aldoc-Venus mutant mice (Figure 4A–F). This allowed us to regard that Venus expression simply mirrors Aldoc expression in the CNS in the mutant mice, particularly in the heterozygotes, of this strain. The present study reconfirmed high levels of Aldoc expression in the retina and in a population of cerebellar PCs and cartwheel cells in the ventral cochlear nucleus, as well as moderate Aldoc expression levels in astrocytes. In the retina, cell type-dependent expression levels were distinguished. In the cerebellum, the striped expression pattern was re-mapped systematically. In addition, Aldoc expression was newly recognized in the inner ear and in the dorsal root ganglion.

### Use of Aldoc-Venus Mice in Relation to the Striped Pattern of Aldoc Expression in the Cerebellum

The striped expression pattern of Aldoc ( = zebrin II) in the cerebellum has been known since the 1980’s [22]. This striped pattern is not homogeneous across lobules, but changes in a certain way from lobule to lobule. It may be convenient to look at it in relation to the longitudinal zonal areas and transverse lobulation when considering how to classify and interpret the striped pattern in the cerebellum. Concerning the longitudinal zonal areas, the stripes are more complex, but also more clearly labeled and more easily traceable, in the vermis than in the pars intermedia and hemisphere. Concerning the transverse lobulation, lobules may be classified into four groups, I–V, VI–VII, VIII–IX, and X, corresponding to AZ, CZ, PZ and NZ, respectively, of Ozol et al. [49]; this classification depends on major differences in the striped pattern of the vermis, although the striped pattern is fairly continuous and does not change abruptly at the boundary between lobule groups [49]. In the pars intermedia, the striped pattern changes significantly between lobules; lobules I–V (AZ) and VIII (PZ) have small number of lightly labeled positive stripes, while lobules VI–VII (CZ) have several clearly labeled stripes. Differences in the striped pattern were also recognized between lobules in the hemisphere, indicating hemispheral extension of transverse zones [50]. Furthermore, it is noticeable that no Aldoc-negative stripe is present in the apex of crus I. We have proposed that this transverse line along the apex of crus I is the “rostrocaudal boundary” of the cerebellar cortex [12], since the projection patterns of climbing and mossy fiber axons can generally be related to this rostrocaudal boundary [16], [26]. All these aspects of the Aldoc striped pattern in the cerebellar cortex were clearly observed in Aldoc-Venus mice in the present study. The striped pattern of Aldoc expression seems to reflect some basic aspects of the organization of the cerebellar cortex. Indeed, the striped pattern originates from the arrangement of PC clusters during development [34] and individual stripes have specific axonal connections to the cerebellar nuclei [11], [17], [47]. Therefore, systematic identification of individual stripes would be beneficial in anatomical and physiological experiments that involve connection-dependent interpretation in any areas of the cerebellar cortex.

Since Aldoc-Venus mice did not show obvious phenotypes in general brain morphology, in the striped pattern in the cerebellum or in behavior, these mice give an appropriate model for such experiments in the cerebellum. By using Aldoc-Venus mice in the present study, we were able to clarify the Aldoc expression pattern in a way more thorough than in previous studies in the entire cerebellar cortex through folia and fissures, and thus, we were able to identify all individual stripes. The results were then mapped in the unfolded scheme of the mouse cerebellar cortex as a revised scheme of the Aldoc stripe pattern. Consequently, stripes became readily identifiable in coronal and horizontal sections of the cerebellum at any levels of sectioning (Figures S2–S5), and also in any aspects of the whole-mount preparation (Figure 6). Those figures can be referred to readily identify Aldoc stripes in coronal and horizontal section, as well as in whole-mount preparation of the mouse cerebellum, in anatomical and physiological experiments.

The Aldoc-Venus mouse can also be used in many further experiments. For example, it is possible to inject tracer or to record neuronal activity in specific Aldoc stripes in *in vivo* and *in vitro* preparations of the cerebellum under fluorescent microscopy in order to facilitate an experimental understanding of the functional significance of the striped organization of the cerebellum. These approaches have been previously undertaken with the post-experimental visualization of stripes with immunostaining [11], [12], [16], [51] and by assuming that the rostral cerebellum is mainly Aldoc-negative and that the caudal cerebellum is mainly Aldoc-positive [21]. In addition to Aldoc, other molecules are also expressed in striped patterns in the cerebellum [19], [33]. The expression patterns of these molecules are either the same as, complementary to, or related in other manners to the pattern of Aldoc expression [18]. Aldoc-Venus mice would facilitate the comparison of expression patterns of these molecules to those of Aldoc throughout the cerebellum.

### Striped Aldoc Expression Pattern in the Flocculus of Aldoc-Venus Mice

The present study showed a striped molecular expression pattern in the rodent flocculus for the first time; expression of Aldoc (and Venus) was much lower in the rostral part (designated Aldoc-negative, 9−) of the flocculus than in its caudal part (designated Aldoc-positive, 9+). We first noticed this in Venus expression in Aldoc-Venus mice, but subsequent Aldoc immunostaining showed that the same pattern also exists in wild type mice (Figure 4E, F). The boundary between these areas was a simple plane parallel to the longitudinal direction of the flocculus.

The reason that this striped pattern was not recognized previously [12], [42] may be a combination of the following: (1) Background peroxidase activity seems to tend to be high in the flocculus (our unpublished observation), which impedes accurate observation of the labeling pattern in immunostaining with diaminobenzidine visualization, (2) the striped pattern in the flocculus cannot be observed in coronal sections, which are usually used to analyze the patterns in other areas of the cerebellum, and (3) the fact that the rostral edge of the paraflocculus is located roughly at the same level as the boundary between the Aldoc-positive and negative stripes in the flocculus (Figure 4E, F) may have hampered recognition of the boundary through human visual perception in horizontal and parasagittal sections.

The present finding that the rostral flocculus is Aldoc-negative in the mouse suggests that the mouse flocculus is comparable to the marmoset flocculus regarding the striped Aldoc expression pattern. The marmoset flocculus has four Aldoc-positive and four Aldoc-negative stripes, all of which are parallel to the axis of the floccular foliation (i.e. the longitudinal plane of the flocculus) [27]; the Aldoc stripes in the mouse were also parallel to the axis of floccular foliation. In the marmoset, the medial flocculus is mostly Aldoc-negative due to the widest Aldoc-negative stripe being located there. The rostral flocculus of the mouse, which is equivalent to the medial flocculus of the marmoset when one considers the rotation of the marmoset flocculus due to the expansion of the hemisphere, was also Aldoc-negative. Thus, both the flocculi have a striped expression pattern of Aldoc that is parallel to the axis of floccular foliation and mostly Aldoc-negative in the rostral (medial in marmoset) flocculus.

The mammalian flocculus is divided into three major, and a few minor, functional compartments, which were explored mainly in rabbit [44], [46] but also in other species including mouse [43]. Aldoc stripes are closely related to cerebellar functional compartments that have different topographic connections of olivocerebellar and PC axonal projections in other parts of the cerebellum [12], [17]. The finding that the flocculus has Aldoc stripes in the Aldoc-Venus mice prompts us to examine the question of how these Aldoc stripes are related to the functional compartments or neural circuits in the flocculus of mice. Although our study was done with indirect techniques and without physiological recordings of neuronal activity, it indicated that the boundaries of the Aldoc stripes were likely to match with the boundaries of functional compartments; Aldoc-positive and -negative stripes seemed to contain the caudal and central functional compartments (zone 1 or HA zone, and zone 2 or VA zone), respectively. However, the whole relationship is not entirely clear yet; particularly, there remains a question of how the two Aldoc stripes (9+ and 9−) harbor as many as three, or more, functional compartments. Also, considering the differences in the striped patterns of Aldoc expression in the flocculus between the mouse and the marmoset, it would be required to study the relationship in different mammalian species to confirm this relationship.

### Functional Significance of Aldoc Expression

Since the striped pattern was virtually normal in the homozygote cerebellar cortex, it seems that Aldoc protein is not involved in the formation of the striped pattern in the cerebellar cortex. Thus, the functional significance of the Aldoc protein expression in relation to the striped pattern of the cerebellum is still not clear.

Aldolase is an enzyme involved in one of the essential steps in glycolysis, a process required in all cells that consume glucose. There are three isozymes, aldolases A, B and C, which are expressed most abundantly in the muscle and in the brain, in the liver, and in the brain, respectively [7], [8]. In the brain, glycolysis occurs mainly in astrocytes, producing lactate, which neurons take up as an energy source through monocarboxylate transporters [52]. Therefore, the expression of Aldoc in astrocytes, and the lack of Aldoc in most types of neurons, is reasonable. The fact that both aldolases A and C are expressed in brain [7], [8] may also explain that the observation that the knock-out of Aldoc expression did not cause any obvious phenotypes in the present study, although detailed expression pattern of aldolase A in the brain has not yet been clarified.

If aldolase A can compensate for Aldoc, how can Aldoc-positive and -negative PCs be functionally different? Aldoc-positive PCs are more likely to survive than Aldoc-negative PCs after acute ischemia [53] or when under chronic pathological conditions, as shown in Niemann-Pick disease type C model mice [54] as well as in other spontaneous mutation mice [55]. These studies have suggested a neuroprotective function of Aldoc. Indeed, Aldoc and/or other aldolases have been also related to non-glycolytic functions, such as direct interactions with vacuolar-H^+^-ATPase [1], [2], neurofilament light chain (NF-L) mRNA [3], F-actin [4], alpha-tubulin [5], dynein [6], and the glucose transporter GLUT4 [56]. It is possible that aldolase itself play a role in PC survival via modulation of vacuolar pH and stabilization of NF-L mRNA; a dysfunction in either will lead to cellular pathology [57], [58]. Although differences in the relative amounts or in the relative affinity to interacting molecules are not known for aldolase A and C in PCs, we speculate that the differences in Aldoc expression level among PCs affects their tolerance for environmental changes. In the retina, human age-related macular degeneration is significantly associated with the presence of anti-Aldoc antibodies [59], presumably leading to the disruption of aldolase functions and to the inflammation in the retina. This suggests that Aldoc-Venus mice may also be useful for the study of molecular mechanisms of various Aldoc functions, including neuroprotective roles, since Aldoc is partially (in heterozygotes) or completely (in homozygotes) knocked out. In this sense, detailed phenotypes of Aldoc-Venus mice are yet to be examined under various situations such as under ischemic and other stress.

## Supporting Information

Figure S1 **Comparison of Aldoc expression and Venus expression levels in the cerebellum among the wild type and mutants.** A–F, Photomicrographs of Venus expression and Aldoc immunostaining in the same section in the wild type (*Aldoc* ^+/+^; A, D), heterozygote (*Aldoc* ^+/Venus^; B, E) and homozygote (*Aldoc* ^Venus/Venus^; C, F). Immunostaining was performed with the same solution and in the same session, and photos were processed with the same exposure and adjustment settings in A–C and D–F. G, Expression of Aldoc and house keeping protein beta-actin in the whole cerebellum examined with Western blotting in wild type, heterozygote and homozygote littermate adult mice. Single Aldoc-specific (about 40 kDa) and beta-actin-specific (42 kDa) bands were recognized. H, Quantitative analyses of protein ratio of Aldoc versus beta-actin by optical densitometry. Ratios were normalized (100%, 47.1% and not detected in the wild type, heterozygote and homozygote samples, respectively). Scale bar in F applies to A–F.
(TIF)

Figure S2 **Aldoc expression pattern in samples of coronal cerebellar sections of an adult Aldoc-Venus mouse, part 1.** Sections are 200 µm separate from one another. Stripes were identified by referring to the results of SSAA (Figure 6). Asterisks indicate stripe 2b+, which was sometime separately located shortly lateral to stripe 2+, in lobule VIa. Dotted lines indicate the rostrocaudal boundary of the cerebellar cortex, where nomenclature for stripes changes [12], [26]. Scale bar in L applies to A–L. See the legends for Figure 1 for abbreviations.
(TIF)

Figure S3 **Aldoc expression pattern in samples of coronal cerebellar sections of an adult Aldoc-Venus mouse, part 2.** Scale bar in U applies to M–U.
(TIF)

Figure S4 **Aldoc expression pattern in samples of horizontal cerebellar sections of an adult Aldoc-Venus mouse, part 1.** Sections are 200 µm separate from one another. Stripes were identified by referring to the results of SSAA (Figure 6). Asterisks indicate stripe 2b+, which was sometime separately located shortly lateral to stripe 2+, in lobule VIa. Dotted lines indicate the rostrocaudal boundary of the cerebellar cortex, where nomenclature for stripes changes [12], [26]. Scale bar in L applies to A–L. See the legends for Figure 1 for abbreviations.
(TIF)

Figure S5 **Aldoc expression pattern in samples of horizontal cerebellar sections of an adult Aldoc-Venus mouse, part 2.** Scale bar in X applies to M–Y.
(TIF)

Figure S6 **Relationship between the expression patterns of HSP25 and Aldoc in the vermis in the Aldoc-Venus mouse.** A–F, Comparison of the Venus expression pattern (A) and the immunostained HSP25 expression pattern (B), which were double labeled in a coronal section of the caudal cerebellum (C, green and magenta, respectively). Images of the squared part under higher magnification are shown in D–F. G–I, SSAA from serial coronal sections of lobules VI–VII and IXc–X. Color channels for Venus (G) and HSP25 (H) are shown separately and also in combination (I, green and magenta, respectively). Asterisks indicate loss of a small number of serial sections at the apices of lobule VII and IXc. J, HSP25 expression pattern mapped on the scheme of Aldoc expression in the caudal vermis. See the legends for Figure 1 for abbreviations.
(TIF)
