# Supplementary figures and images for: Detailed Expression Pattern of Aldolase C (Aldoc) in the Cerebellum, Retina and Other Areas of the CNS Studied in Aldoc-Venus Knock-In Mice
Source: PLoS One. 2014 Jan 27;9(1):e86679. doi: 10.1371/journal.pone.0086679 (PMC3903578; doi:10.1371/journal.pone.0086679)

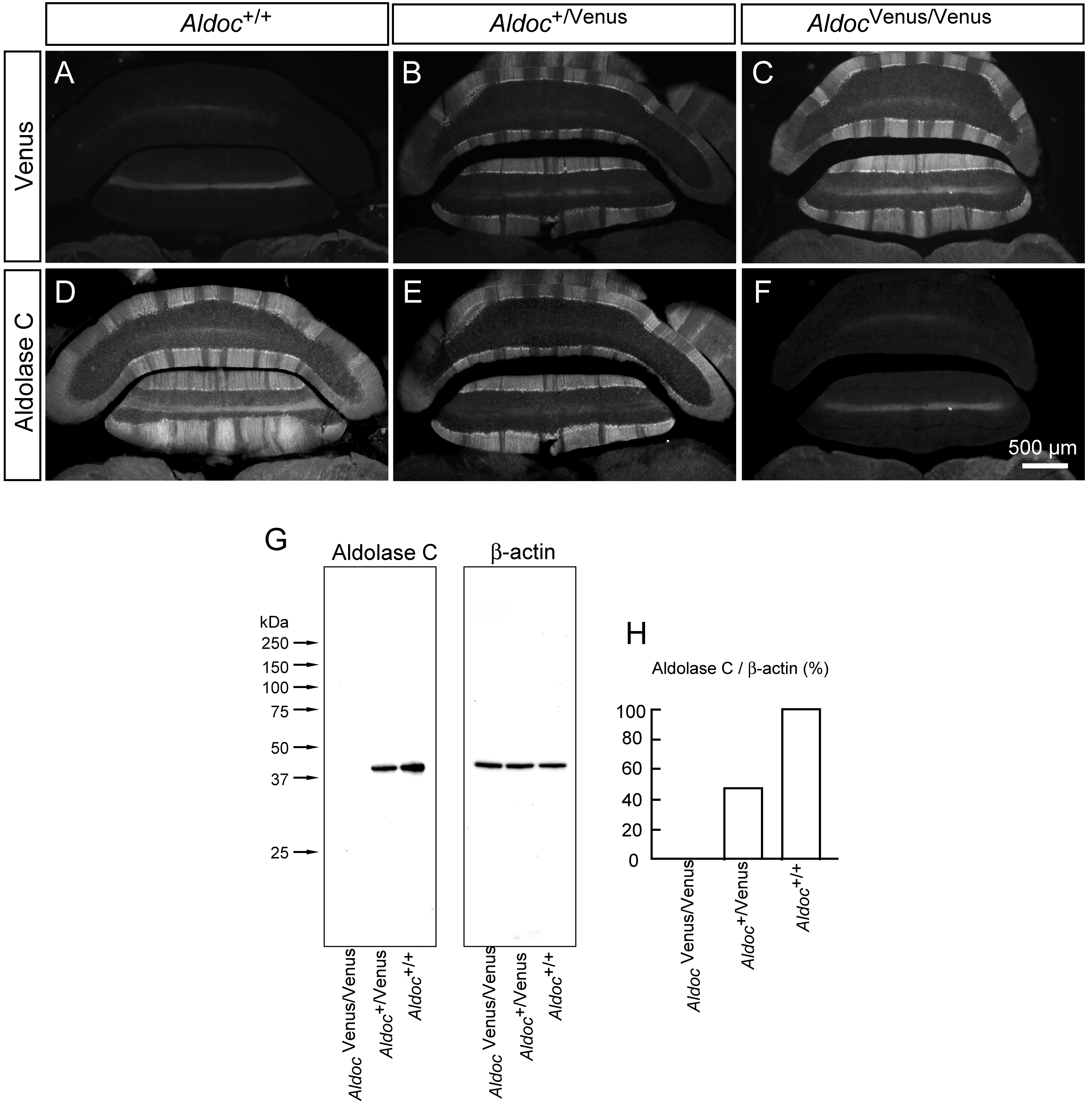

Supplement: Figure S1 — Comparison of Aldoc expression and Venus expression levels in the cerebellum among the wild type and mutants. A–F, Photomicrographs of Venus expression and Aldoc immunostaining in the same section in the wild type (Aldoc +/+; A, D), heterozygote (Aldoc +/Venus; B, E) and homozygote (Aldoc Venus/Venus; C, F). Immunostaining was performed with the same solution and in the same session, and photos were processed with the same exposure and adjustment settings in A–C and D–F. G, Expression of Aldoc and house keeping protein beta-actin in the whole cerebellum examined with Western blotting in wild type, heterozygote and homozygote littermate adult mice. Single Aldoc-specific (about 40 kDa) and beta-actin-specific (42 kDa) bands were recognized. H, Quantitative analyses of protein ratio of Aldoc versus beta-actin by optical densitometry. Ratios were normalized (100%, 47.1% and not detected in the wild type, heterozygote and homozygote samples, respectively). Scale bar in F applies to A–F. (TIF) [file pone.0086679.s001.tif]

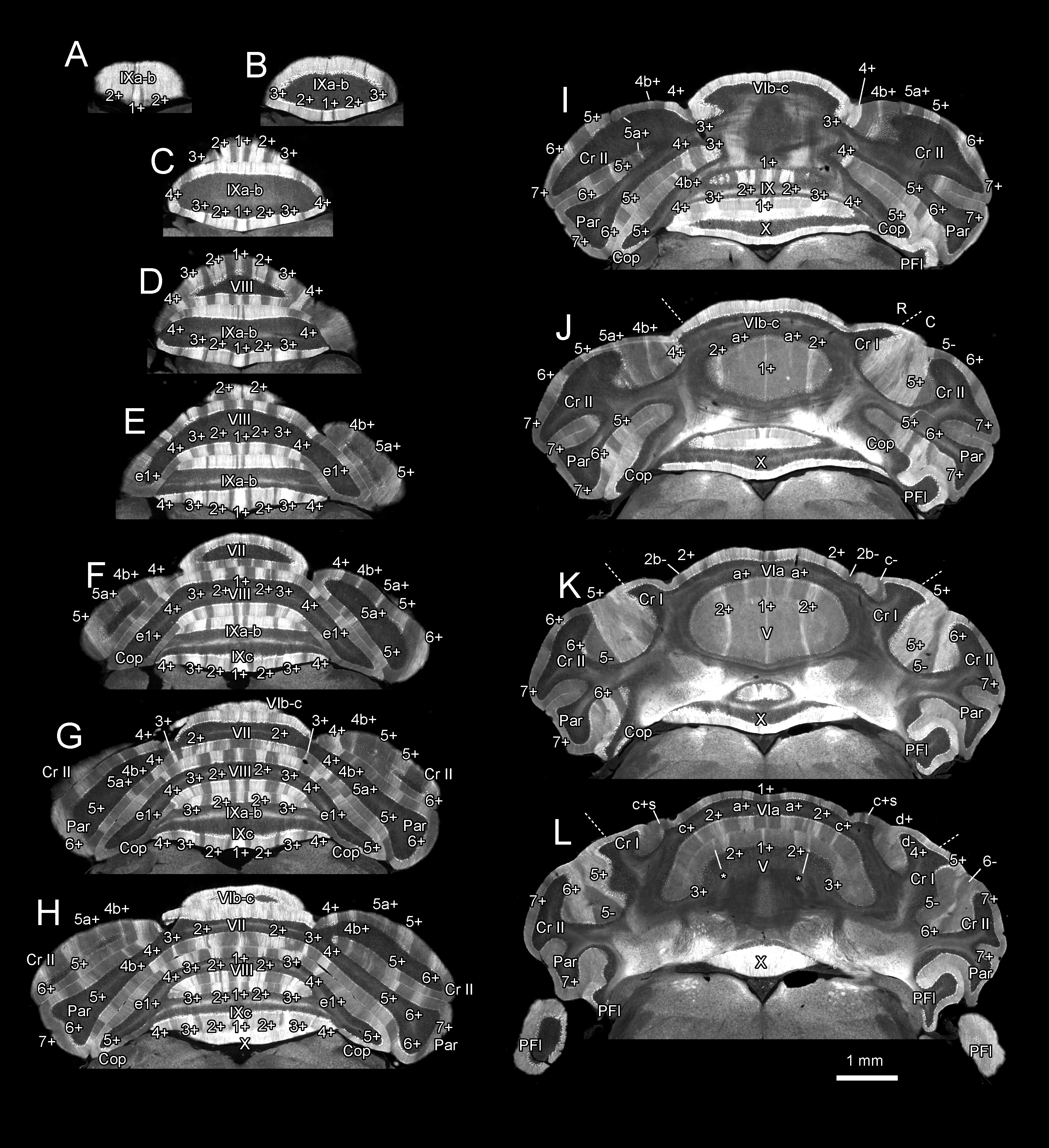

Supplement: Figure S2 — Aldoc expression pattern in samples of coronal cerebellar sections of an adult Aldoc-Venus mouse, part 1. Sections are 200 µm separate from one another. Stripes were identified by referring to the results of SSAA (Figure 6). Asterisks indicate stripe 2b+, which was sometime separately located shortly lateral to stripe 2+, in lobule VIa. Dotted lines indicate the rostrocaudal boundary of the cerebellar cortex, where nomenclature for stripes changes [12], [26]. Scale bar in L applies to A–L. See the legends for Figure 1 for abbreviations. (TIF) [file pone.0086679.s002.tif]

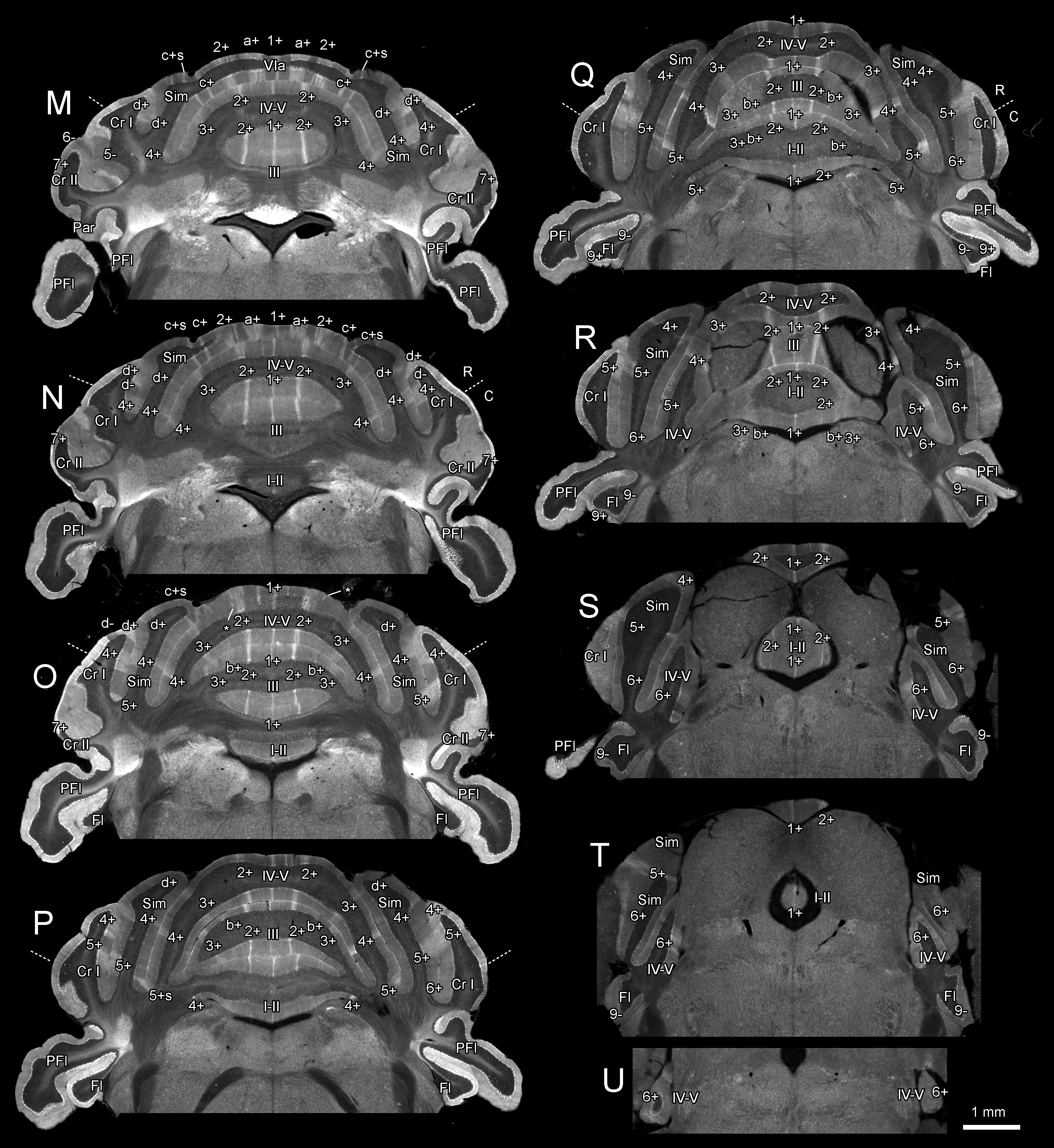

Supplement: Figure S3 — Aldoc expression pattern in samples of coronal cerebellar sections of an adult Aldoc-Venus mouse, part 2. Scale bar in U applies to M–U. (TIF) [file pone.0086679.s003.tif]

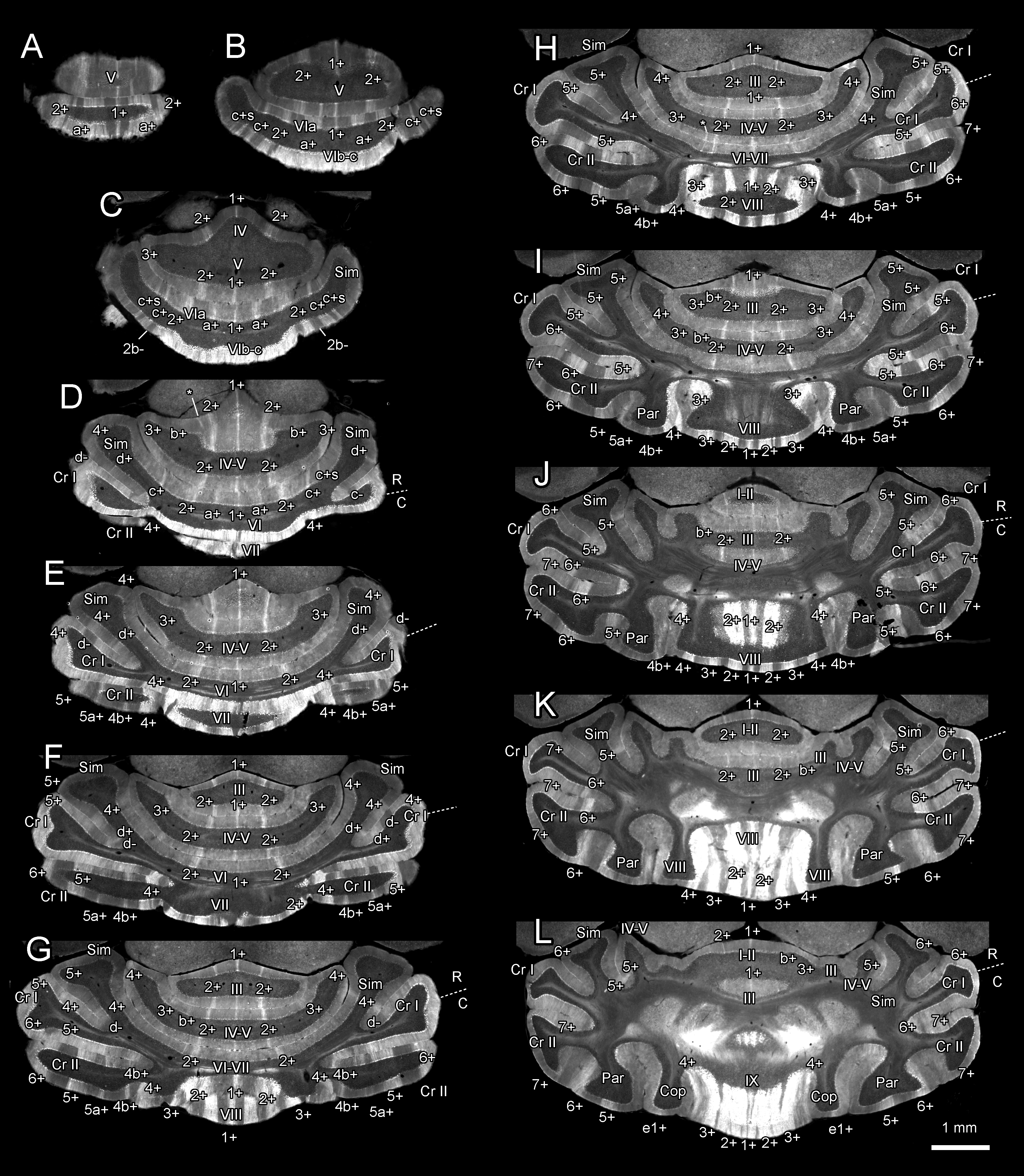

Supplement: Figure S4 — Aldoc expression pattern in samples of horizontal cerebellar sections of an adult Aldoc-Venus mouse, part 1. Sections are 200 µm separate from one another. Stripes were identified by referring to the results of SSAA (Figure 6). Asterisks indicate stripe 2b+, which was sometime separately located shortly lateral to stripe 2+, in lobule VIa. Dotted lines indicate the rostrocaudal boundary of the cerebellar cortex, where nomenclature for stripes changes [12], [26]. Scale bar in L applies to A–L. See the legends for Figure 1 for abbreviations. (TIF) [file pone.0086679.s004.tif]

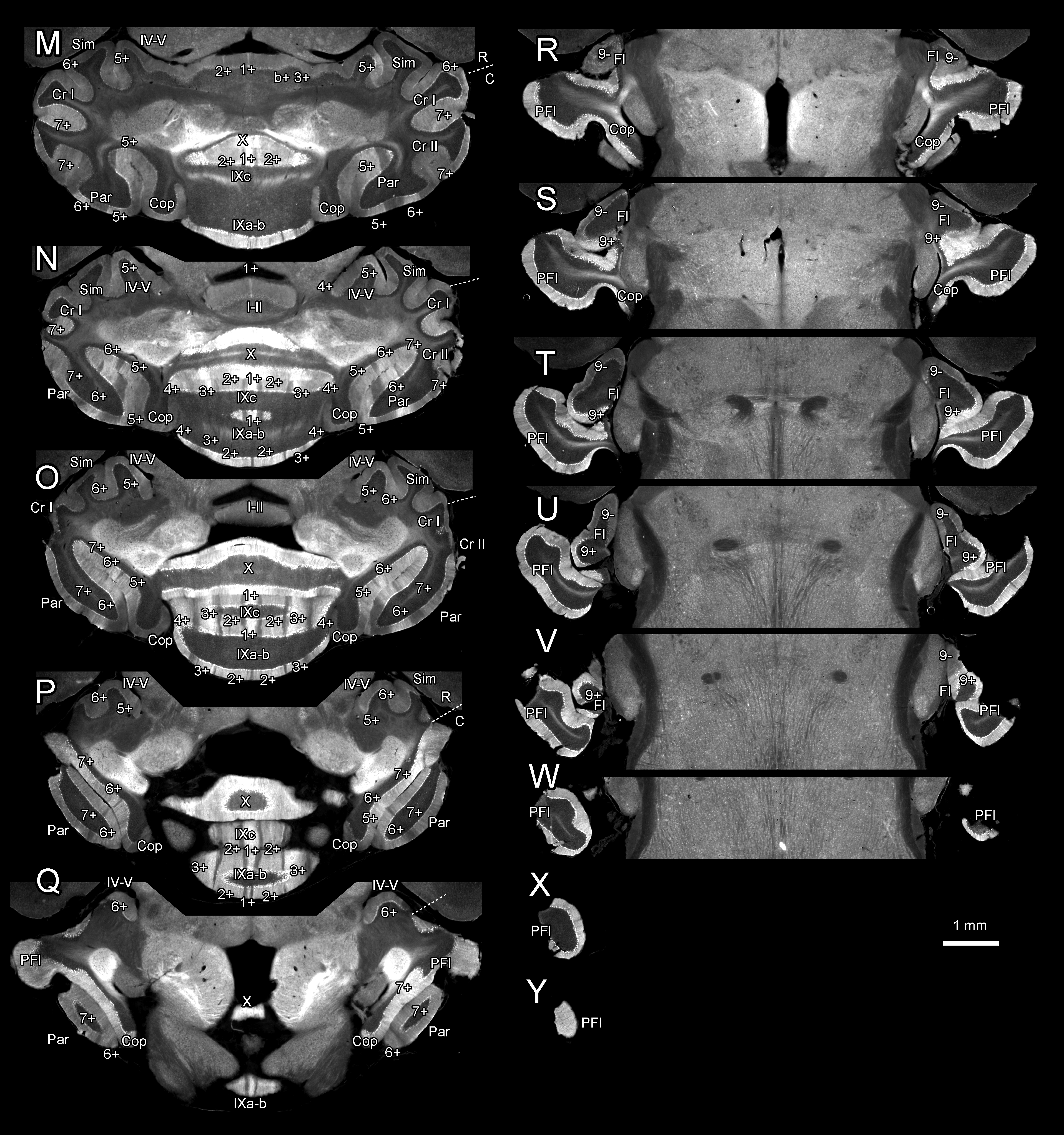

Supplement: Figure S5 — Aldoc expression pattern in samples of horizontal cerebellar sections of an adult Aldoc-Venus mouse, part 2. Scale bar in X applies to M–Y. (TIF) [file pone.0086679.s005.tif]

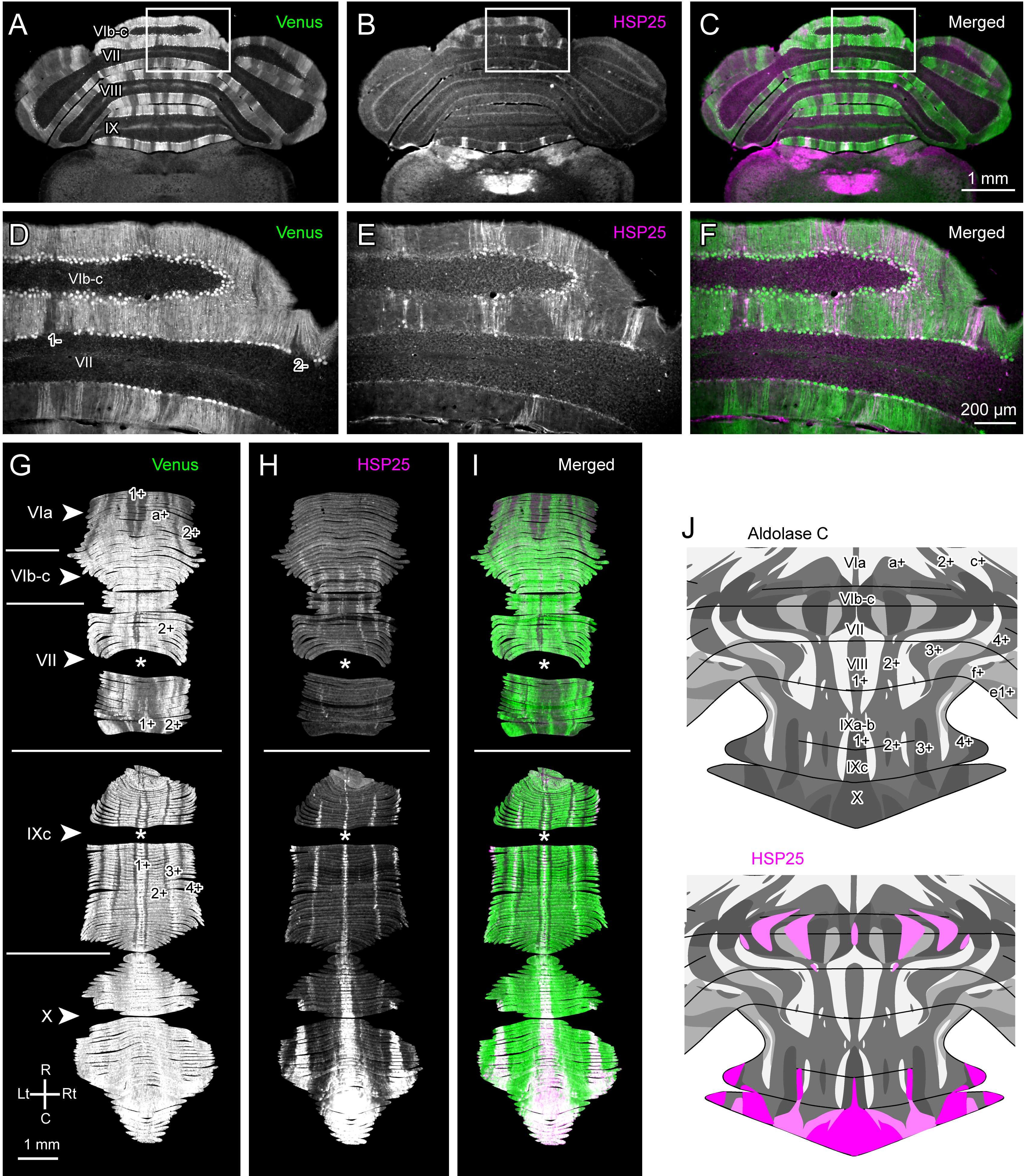

Supplement: Figure S6 — Relationship between the expression patterns of HSP25 and Aldoc in the vermis in the Aldoc-Venus mouse. A–F, Comparison of the Venus expression pattern (A) and the immunostained HSP25 expression pattern (B), which were double labeled in a coronal section of the caudal cerebellum (C, green and magenta, respectively). Images of the squared part under higher magnification are shown in D–F. G–I, SSAA from serial coronal sections of lobules VI–VII and IXc–X. Color channels for Venus (G) and HSP25 (H) are shown separately and also in combination (I, green and magenta, respectively). Asterisks indicate loss of a small number of serial sections at the apices of lobule VII and IXc. J, HSP25 expression pattern mapped on the scheme of Aldoc expression in the caudal vermis. See the legends for Figure 1 for abbreviations. (TIF) [file pone.0086679.s006.tif]
